# Bacterial RNA-free RNase P: Structural and functional characterization of multiple oligomeric forms of a minimal protein-only ribonuclease P

**DOI:** 10.1016/j.jbc.2023.105327

**Published:** 2023-10-06

**Authors:** Catherine A. Wilhelm, Leena Mallik, Abigail L. Kelly, Shayna Brotzman, Johnny Mendoza, Anna G. Anders, Suada Leskaj, Carmen Castillo, Brandon T. Ruotolo, Michael A. Cianfrocco, Markos Koutmos

**Affiliations:** 1 Department of Chemistry, University of Michigan, Ann Arbor, Michigan, USA; 2 Center for Computational and Genomic Medicine and Department of Pathology and Laboratory Medicine, Children's Hospital of Philadelphia, Philadelphia, Pennsylvania, USA; 3 Department of Biological Chemistry, University of Michigan, Ann Arbor, Michigan, USA; 4 Life Sciences Institute, University of Michigan, Ann Arbor, Michigan, USA; 5 Program in Biophysics, University of Michigan, Ann Arbor, Michigan, USA

**Keywords:** tRNA processing, endonuclease, RNase P, precursor tRNA (pre-tRNA), structural biology, nucleic acid enzymology

## Abstract

tRNAs are typically transcribed with extended 5′ and 3′ ends that must be removed before they attain their active form. One of the first steps of tRNA processing in nearly every organism is the removal of the 5′ leader sequence by ribonuclease P (RNase P). Here, we investigate a recently discovered class of RNase P enzymes, Homologs of *Aquifex* RNase P (HARPs). In contrast to other RNase Ps, HARPs consist only of a metallonuclease domain and lack the canonical substrate recognition domain essential in other classes of proteinaceous RNase P. We determined the cryo-EM structure of *Aquifex aeolicus* HARP (Aq880) and two crystal structures of *Hydrogenobacter thermophilus* HARP (Hth1307) to reveal that both enzymes form large ring-like assemblies: a dodecamer in Aq880 and a tetradecamer in Hth1307. In both oligomers, the enzyme active site is 42 Å away from a positively charged helical region, as seen in other protein-only RNase P enzymes, which likely serves to recognize and bind the elbow region of the pre-tRNA substrate. In addition, we use native mass spectrometry to confirm and characterize the previously unreported tetradecamer state. Notably, we find that multiple oligomeric states of Hth1307 are able to cleave pre-tRNAs. Furthermore, our single-turnover kinetic studies indicate that Hth1307 cleaves pre-tRNAs from multiple species with a preference for native substrates. These data provide a closer look at the nuanced similarities and differences in tRNA processing across disparate classes of RNase P.

tRNAs play a key role in biology, shuttling amino acids into the ribosome during protein synthesis. Like other RNAs in the translation machinery, tRNAs are transcribed as precursors that must be matured before they attain their biologically active form. Precursor tRNA (pre-tRNA) maturation often includes the removal of 5′ leader and 3′ trailer sequences and/or introns in the anticodon stem loop, as well as the addition of nucleoside modifications (Fig. 1*A*). The removal of 5′ leader sequences by ribonuclease P (RNase P) is one of the most highly conserved steps in tRNA maturation across all domains of life (1, 2). RNase P is an endoribonuclease that is essential in all organisms, with the exception of *Nanoarchaeum equitans*, which does not encode 5′ leader sequences in its tRNA (3, 4, 5, 6).

**Figure 1:**
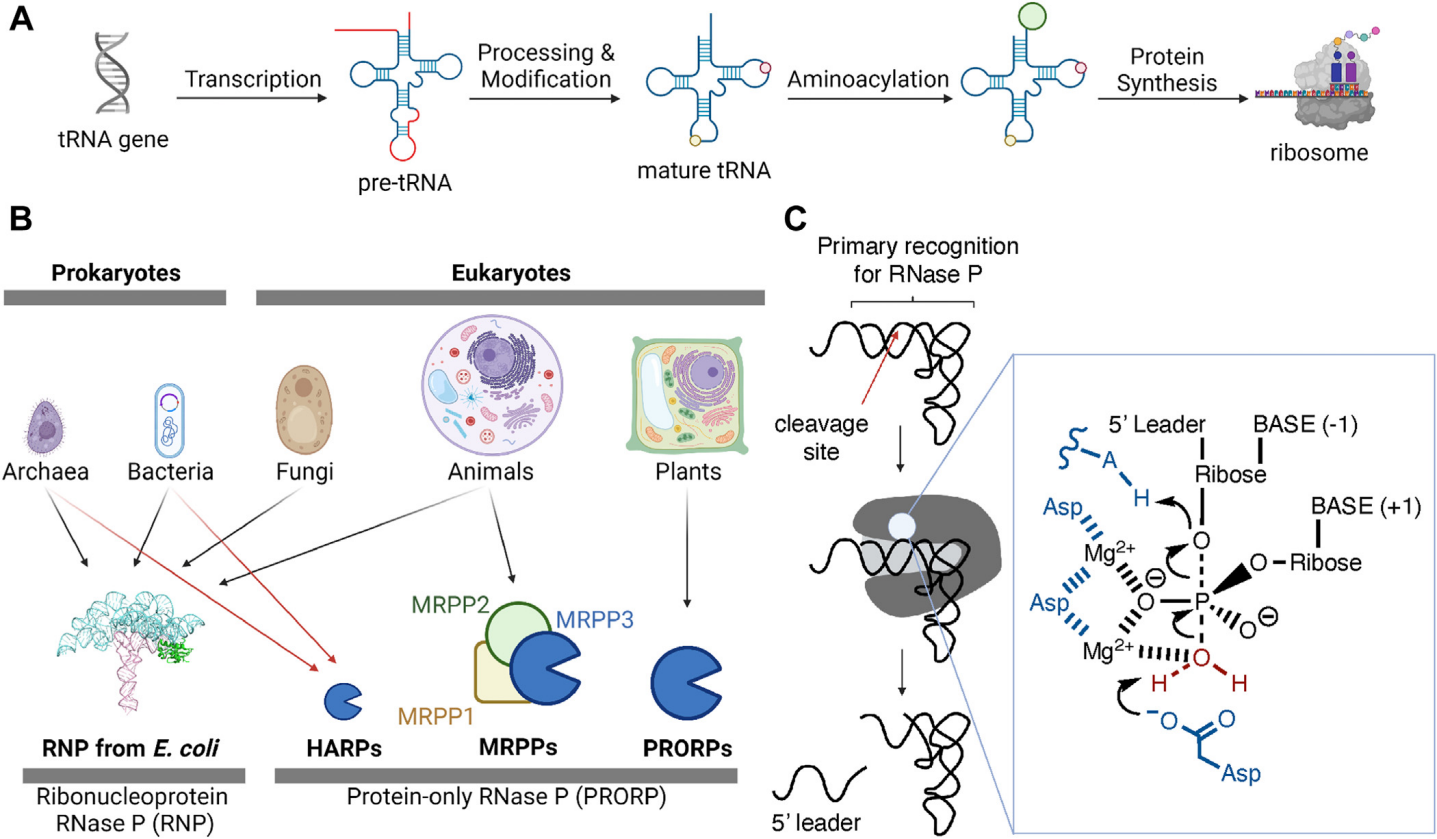
**An overview of RNase P.***A*, tRNA transcripts undergo processing, modification, and aminoacylation prior to use in translation. *B*, RNase P exists in all domains of life in different forms, including ribonucleoprotein (RNP) complexes and protein only RNase P (PRORP) enzymes. PRORPs can be further categorized into the monomeric PRORP found in plants, the PRORP complex found in mitochondria (MRPP), and the HARP homo-oligomer found in bacteria and archaea. *C*, the conserved RNase P mechanism includes substrate recognition based on the 42 Å distance between the tRNA elbow and 5′ cleavage site, followed by divalent cation-mediated hydrolysis of the 3′-5′ phosphate bond between the nucleotides at position −1 and +1. HARP, Homologs of Aquifex RNase P.

RNase P has evolved independently multiple times, leading to a diverse set of RNase P molecular architectures across different species (Fig. 1*B*) (7). The ribonucleoprotein (RNP) form of RNase P was one of the first ribozymes discovered and has been studied for decades. This molecule consists of a catalytic RNA core associated with a varying number of accessory proteins (2, 8, 9, 10). However, RNase P is not always a ribozyme; proteinaceous, RNA-free forms of RNase P (termed protein-only RNase P, or PRORP) exist in all eukaryotes except Amoebozoa (11, 12, 13). PRORPs are single proteins with a catalytic metallonuclease domain, a central linker domain, and a pentatricopeptide repeat substrate recognition domain (14). In metazoans, the mitochondrial form of the enzyme requires two additional protein partners to function *in vivo* (11).

Despite these major structural differences, RNase P enzymes have a conserved catalytic strategy. Catalysis is carried out by a divalent magnesium ion that hydrolyzes the phosphodiester bond between nucleotides at positions −1 and +1 of pre-tRNAs (Fig. 1*C*). This results in the formation of a tRNA with a mature 5′ end with a phosphate group and a leader sequence with a 3′ hydroxyl group. In all RNP and PRORP RNase P enzymes characterized thus far, the active site is approximately 42 Å from the binding region. In PRORP, the binding region consists of positively charged amino acids that bind to the negatively charged phosphate backbone of the elbow region of the substrate pre-tRNA (15). This 42 Å distance is attributed to being a “molecular ruler” for all RNP and PRORP RNase P that measures from the T and D loops in the elbow region of the pre-tRNA to the cleavage site between the +1 and −1 nucleotides, a distance which is conserved among tRNAs (16).

Recently, a distinct class of PRORPs were discovered in the thermophilic bacterium *Aquifex aeolicus* (17). The RNase P that was discovered in *A. aeolicus* is vastly different from previously characterized PRORPs. In contrast to other PRORPs, the *A. aeolicus* enzyme is quite small (23 kDa) and only shares sequence similarity with a single region of other PRORPs (17), the metallonuclease domain. Additional homologs of this small RNase P enzyme were identified in numerous bacterial and archaeal species, and these proteins are collectively referred to as Homologs of *Aquifex* RNase P (HARPs) (17).

The lack of an apparent substrate recognition domain in HARPs raised questions about how HARP enzymes interact with their pre-tRNA substrates. Initial research established that HARP from *A. aeolicus* exists as a large homo-oligomeric complex (17). To understand how this oligomerization may contribute to substrate recognition, the cryo-EM structure of a homo-dodecameric (12-mer) HARP assembly has been published by three independent groups. These 12-mer structures include HARP from *A. aeolicus* (Aq880) (18), *Halorhodospira halophila* SL1 (Hhal2243) (19), and *Thermocrinis ruber* (20). HARP structures in additional oligomeric states have also been published, including HARP dimers from *Planctomyces bacterium* and *Thermococcus celer* and a HARP tetramer from *P. bacterium* complexed with *Escherichia coli* pre-tRNA^His^ (20). These data all show that a monomer of HARP is a VapC-like endonuclease that consists of a PIN domain-like metallonuclease domain and two protruding α helices, which are thought to be responsible for substrate recognition (18, 19). The active site is conserved among HARPs and PRORPs, with five negatively charged residues, predominantly aspartate, positioned to coordinate two catalytic magnesium ions. The exact role of HARP enzymes *in vivo* remains to be elucidated, as most organisms that encode HARP also encode the RNP RNase P, and deletion of HARP in *M. mazei* and *H. volcanii* had no effect on their growth compared to the WT strains (21).

The work presented here uses a combination of cryo-EM and X-ray crystallography to characterize three homo-oligomeric states of HARP: a tetramer, a dodecamer, and a novel tetradecamer (14-mer). We also provide single turnover kinetic data demonstrating that *Hydrogenobacter thermophilus* HARP (Hth1307), which is mostly tetradecamer, has a strong preference for its native pre-tRNA substrates. Our studies allow us to propose an alternative binding model for HARP enzymes and highlight the remaining questions in the field.

## Results

### Aq880 and Hth1307 HARP occupy different oligomeric states

We concurrently worked on Aq880 and Hth1307 HARPs, both of which expressed and purified as a mixture of oligomers. When purifying Aq880, we observed tetramers and dodecamers that were difficult to separate. Single particle analysis with cryo-EM was used to investigate the structure of Aq880 as it is uniquely suited to deconvoluting mixtures. Close inspection of the 2D class averages reveals orientation bias in our dodecamer particles (Fig. S1). From this data, we were able to create a low-resolution model of the Aq880 dodecamer (Fig. S1), which agrees with the radial arrangement of dimers seen in previously published structures (18, 19).

Next, we focused our attention on Hth1307, whose multiple oligomeric states were more easily separated during purification. The predominant product was a large oligomeric assembly, although there was also a small proportion of enzyme that purified as a smaller oligomeric assembly. These isolated fractions were used to successfully grow protein crystals of Hth1307 in two different oligomeric states, described below. The large Hth1307 assembly eluted at a lower elution volume than that of Aq880 on the elution profile during size-exclusion chromatography (SEC), indicative of a higher molecular weight and oligomeric state (Fig. S2, *A* and *B*).

**Figure 2:**
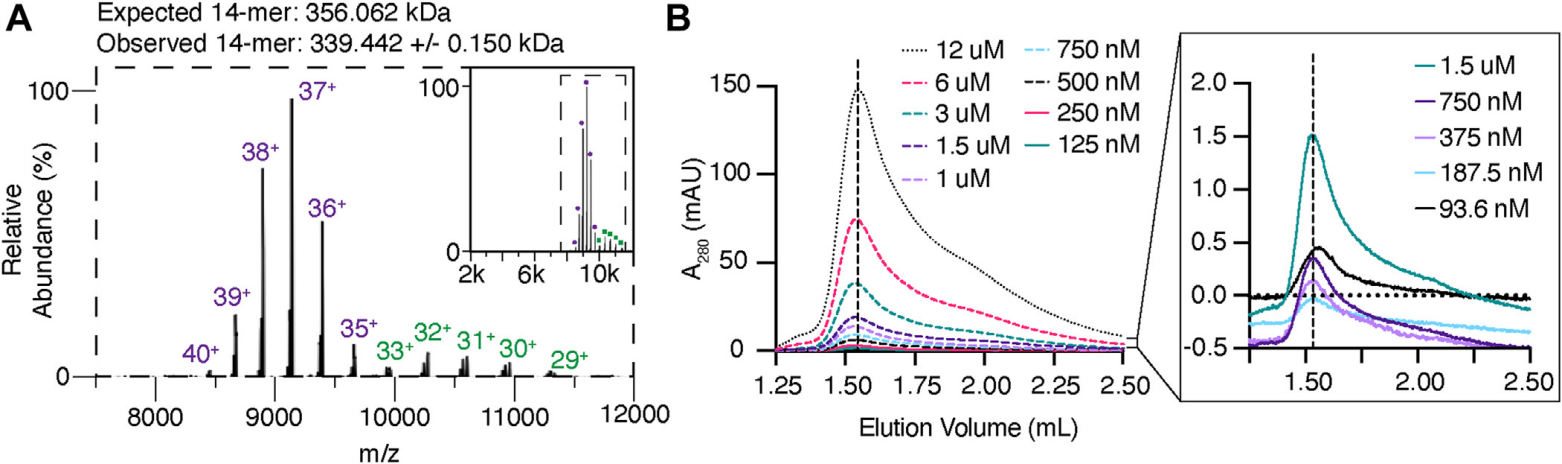
**Native MS and SEC demonstrating oligomeric states of HARP from *Hydrogenobacter thermophilus*.***A*, native mass spectra were obtained using His-tagged Hth1307. No oligomeric states below or above a 14-mer are observed. Differences in the expected and observed molecular weights are due to partial loss of labile His-tags. Two charge state series (or charge envelopes) are observed for the 14-mer species (*purple circles* and *green squares*) indicative of two main conformational states. The high charge state series is shown in *purple* and the low charge state series is shown in *green*. A detailed and zoomed view of this data can be found in Fig. S5. *B*, size-exclusion chromatography was conducted using Hth1307 samples ranging from 93.6 nM to 12 μM as indicated in the graph legend, while monitoring the absorbance at 280 nm to look for changes in the molecular weight of the complex *via* the elution volume. A size-exclusion profile using standard molecular weight proteins can be found in Fig. S2. A260 levels lower than the A280 confirmed a lack of RNA contamination (Fig. S4). The inset is zoomed in to a lower absorbance range. HARP, Homologs of Aquifex RNase P; MS, mass spectrometry.

Native mass spectrometry (MS) with nano-electrospray ionization was used to quantify the oligomeric populations of Hth1307. This method separates proteins and protein complexes by size, shape, and charge prior to MS analysis, providing accurate and detailed mass measurements and stoichiometry information (22). Experiments using His-tagged Hth1307 revealed a single oligomeric state of a tetradecamer (14-mer, Figs. 2*A* and S3). While there is a difference in the observed and expected molecular weights of the 14-mer, these differences were identified to be a result of labile His-tags (23). Specifically, an average of 5 to 6 tags were lost during MS analysis. In the mass spectra, two charge envelopes of the 14-mer can be readily observed. While the high charge states could be representative of a more unfolded 14-mer due to coulombic repulsion (24, 25, 26), this bimodality potentially indicates two predominant conformational states that were previously hypothesized (18, 20). Prior to this point, the highest oligomeric state of HARP proteins was shown to be a dodecamer (12-mer).

Because native MS is limited to larger concentrations of protein (500–1000 nM), these experiments were accompanied by SEC experiments to monitor the elution volume, which corresponds to molecular weight, of Hth1307 at a variety of lower concentrations more similar to the assay conditions described below. There was no observed shift in the elution volume of the major population at 1.54 ml when starting with concentrations ranging from 94 nM to 126 μM (Fig. 2*B*). Combined, these results demonstrate that while Hth1307 exists in many oligomeric states in solution, the majority of the sample is a tetradecamer (14-mer).

### The X-ray structure of the Hth1307 tetramer reveals the minimal functional protein assembly in substrate-free form

**Figure 3:**
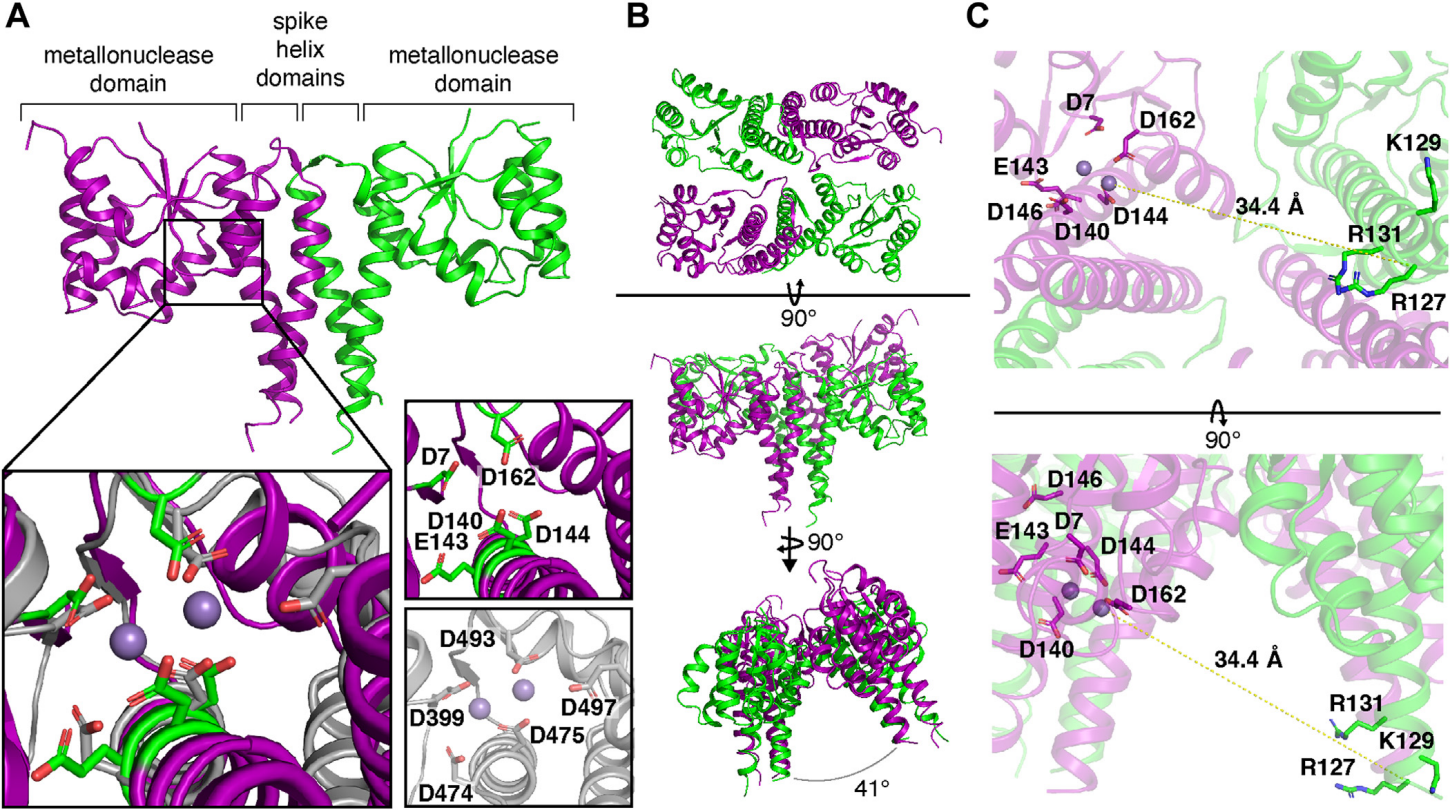
**Crystal structure of the Hth1307 tetramer.***A*, one monomer shown in *purple* and one in *green*. The inset zooms in on the active site of Hth1307 (active site residues shown in *green*) aligned to that of At PRORP1 (4G24, *gray*). The divalent cations (Mn^2+^) from the At PRORP1 structure are shown in *light purple*. *B*, generation of crystal symmetry mates for a Hth1307 dimer in the asymmetric unit reveals the captured structure is a tetramer, shown here in three views. *C*, the distance between the Mn^2+^ ions from At PRORP1 to the C_α_ of residue R127 of Hth1307 was used to approximate the distance between the active site of one monomer and the proposed binding site in the opposing monomer, which is 34.4 Å. The divalent cations were modeled based on the At PRORP1 structure. Hth1307, *Hydrogenobacter thermophilus* HARP; PRORP, protein-only RNase P.

We sought to better understand the structure and function of Hth1307 using protein crystallography. From the lower molecular weight fraction of Hth1307 discussed above, we first determined the crystal structure of Hth1307 to 2.5 Å as a dimer within the asymmetric unit (Fig. 3*A* and Table S1). The homotetrameric Hth1307 assembly is generated through symmetry by two identical dimers resulting in a dimer of dimers (Fig. 3*B*). Each monomer in the Hth1307 dimer consists of a PIN-like metallonuclease domain and a spike helix (SH) domain.

The metallonuclease domain consists of an α/β/α domain with a central four-stranded parallel β-sheet (Fig. S5*A*). Alignment of the Hth1307 metallonuclease domain with that of At PRORP1 (14) demonstrates a conserved active site with five negatively charged residues positioned to coordinate two divalent metal ions (Fig. 3*A*, inset) (27). Although Hth1307 does not have a residue directly comparable to D497 in At PRORP1, D144 in Hth1307 is positioned in a manner that may fill a similar role upon conformational rearrangement. However, as of yet, there are no structures of HARP with a metal ion properly chelated for catalysis nor have mechanistic studies established if HARP requires two metal ions for endonuclease activity as shown in *At* PRORP studies (27).

The SH domain is a protruding α-helical domain consisting of helices α6 and α7 (Figs. 3*A* and S5*B*), which substantially contribute to the dimerization interface by forming an extensive four-helical bundle. The dimer interface is further stabilized by interactions highlighted in Figs. S5*B* and S6 and has a total surface area of 1459.3 Å^2^ as calculated by PISA (28). In addition to the predominantly hydrophobic interactions, electrostatic interactions include hydrogen bonding between β4 of each monomer, hydrogen bonding between Y87 and D146′ in the SH domain, and salt bridges in the SH domain between residues E105 and R131′ and residues E105′ and R127 (Figs. S5*B* and S6).

In the tetramer, two dimers come together in a side-by-side arrangement, where the two dimers are angled at 41° (Fig. 3*B*). The interface is mostly formed by contacts between the C-terminus of α5 through the N-terminus of α6 of one protomer (residues 63–77) and the C-terminus of α8 through the C-terminus of α9 in the opposing protomer (residues 170–189, Figs. S5*B* and S6). The tetramer interface is considerably smaller than the dimer interface, with a surface area of 589.5 Å^2^ between monomers, for a total of 1179.0 Å^2^ across the entire interface (Table S2) (28). This interface has only two salt bridges, both between R75 and D170′, although there are a number of other residues that participate in backbone and side chain hydrogen bonding, hydrophobic interactions, and pi–pi stacking (Figs. S5*C* and S6. As seen in Fig. S6, most of the residues involved in the dimer interface are conserved, likely to preserve the interface.

Tetramer formation has been proposed to be important for pre-tRNA binding (19, 20). Both RNP and PRORP enzymes recognize the tRNA elbow partly through shape complementarity. Both the RNPs and PRORPs form a large cleft spanning ∼42 Å from the active site to an RNA-binding region, which is wide enough to accommodate the entire length of the tRNA acceptor stem (16). The 5′ end of the acceptor stem is positioned in the metal active site, and the T and D loops are recognized by the pentatricopeptide repeat domain in PRORP (29, 30, 31) and MRPP3 (32) and the CR-II/CR-III modules in human and bacterial RNP RNase P (33, 34). This arrangement allows RNase Ps to recognize tRNA structural elements and select substrates through a molecular ruler mechanism (29, 33, 34, 35, 36, 37). Upon inspection of the electrostatic surface of the tetramer, there is a positively charged region of the SH domain (Fig. S7, (38)) that includes residues R125, R127, K129, and R131 (Fig. 3*C*), which have been confirmed to be necessary for pre-tRNA processing activity (19). These residues are highly conserved across HARPs from different species. In this structure, the positively charged region is 34.4 Å from the active site of the neighboring dimer (Fig. 3*C*), which is not wide enough to accommodate the canonical structure of tRNA without any large protein conformational rearrangement. Therefore, the conformation captured in this crystal structure is unlikely to be the conformation that supports substrate binding and nuclease activity. However, as discussed later in this paper, kinetic experiments demonstrate the protein we isolated maintains nuclease cleavage activity, possibly through substrate-induced conformational changes that have yet to be captured or characterized.

### Structural characterization of the Hth1307 tetradecamer using crystallography

**Figure 4:**
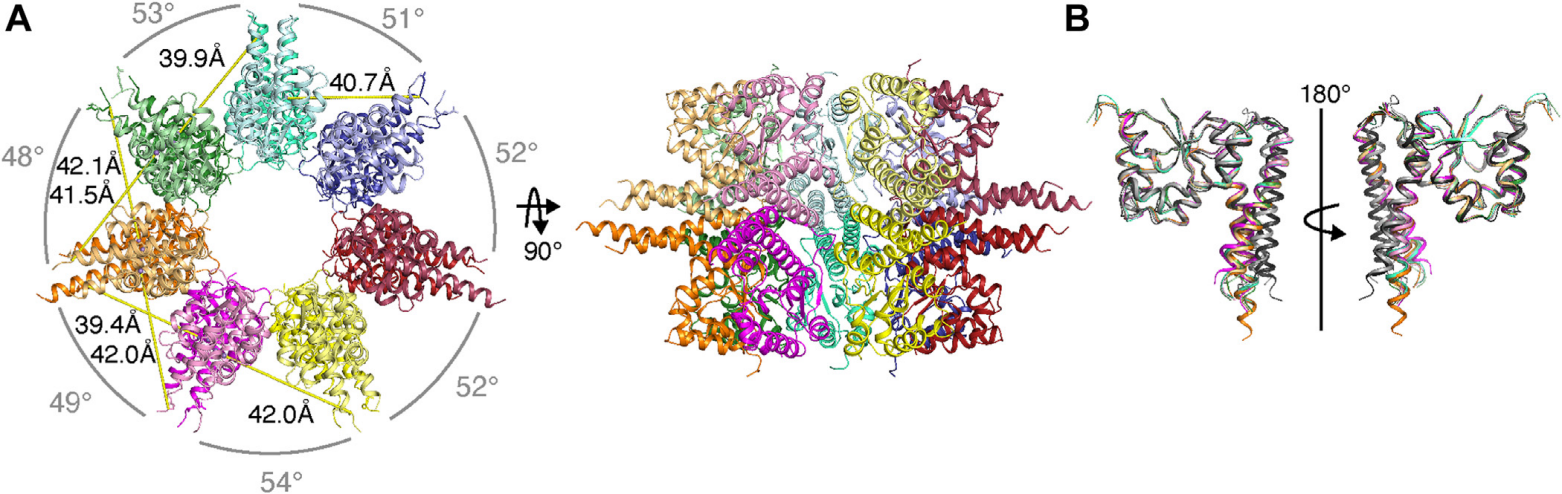
**Crystal structure of the Hth1307 tetradecamer.***A*, top view of the Hth1307 14mer. Measurements from the divalent cation in the active site modeled based on alignment with At PRORP1 (4G24) to R125 in the putative binding site were calculated in PyMOL. Rotation of the complex by 90° along the *x*-axis gives a side view that shows the complex is flat with no gaps between tetramer interfaces. *B*, alignment of unique monomers from the Hth1307 tetramer and tetradecamer structures was performed in PyMOL, with RMSD values ranging from 0.167 to 1.158 Å over 1036 to 1076 atoms. The two monomers in *gray* correspond to those from the Hth1307 tetramer structure, and the other seven monomers have the same coloring shown in (*A*). Hth1307, *Hydrogenobacter thermophilus* HARP; PRORP, protein-only RNase P.

The high-molecular-weight fraction of Hth1307 produced crystals under different conditions than those of the tetramer described above. The larger unit cell of these crystals implied a larger oligomeric formation. Molecular replacement revealed a heptamer in the asymmetric unit that, upon generation of its symmetry mate, produces a tetradecamer (14-mer) flat cylindrical ring consisting of a radial arrangement of seven HARP dimers (Fig. 4*A* and Table S1). The structure was solved to 3.2 Å resolution. We aligned the seven unique monomers from the 14-mer to both of the unique monomers from our tetramer structure (Fig. 4*B*). This demonstrates that all of the metallonuclease domains in our Hth1307 tetramer and 14-mer structures are nearly identical, with RMSD values ranging from 0.87 to 1.16 Å over 1021 to 1088 atoms. Notably, the dimer and tetramer interfaces of the 14-mer are fairly similar when compared to our Hth1307 tetramer structure (Fig. S6). The angles between dimers in the 14-mer structure range from 48° to 54°, and the distances between the proposed tRNA elbow binding region and the active site ranges from 39.4 to 42.1 Å (Fig. 4*A*). The dimer and tetramer interfaces in the tetradecamer have average surface areas of 992.3 Å^2^ and 501.1 Å^2^, respectively (Table S2), similar to the interface surface areas seen in the tetramer structure. In addition, the hydrogen bonding and salt bridges of the interfaces are formed by the same amino acids in both structures (Fig. S6).

The tetradecamer presented here is the largest oligomeric HARP assembly to be described so far. Similar to the smaller HARP dodecamers, the Hth1307 tetradecamer forms radial assemblies. Despite the additional dimer compared to the dodecamers, the Hth1307 tetradecamer maintains a roughly 42 Å distance from the active site to the proposed binding site. The next section will closely address the similarities and differences of all HARP oligomeric structures.

### Structural comparison of high order HARP oligomers

Structure alignments were used to compare our structures of Hth1307 (this work) to Hhal2243 (7OG5 (19)), Aq880 (7F3E (18)), TcHARP (7E8J (20)), and PbHARP (7E8K and 7E8O (20)) using PyMOL (pymol.org), shown in Fig. S8. Accompanying sequence alignments with secondary structure information can be found in Fig. S6. Comparison of the residues involved in the dimer and tetramer interfaces using PISA (28) demonstrates that the interactions are predominantly hydrophobic. Our Hth1307 structures have conformations that most closely resemble 7OG5, 7E8K, and 7E8J, which have a continuous α7 helix in the SH domain, rather than 7F3E and 7E8O, which have a split α7 helix due to the rearrangement of a conserved GI(I/L)DS motif that may better orient D140 for metal ion binding in the active site.

When comparing higher-order HARP complexes, there is a significant deviation of our structure compared to previously solved structures, as seen in Fig. S9. The published models of dodecameric HARP adopt a left-handed helical structure that causes the dimers involved in the closing interface to be staggered by approximately 20 Å. Strikingly, unlike all of the dodecameric structures, our tetradecameric structure has no helical features and is instead a flat ring with nearly identical tetramer interfaces throughout. It is unclear whether the helical structures or the flat ring structure are biologically relevant or required for activity.

### Hth1307 prefers native substrates compared to non-native substrates

We investigated the substrate preference of the Hth1307 protein that we structurally characterized by assessing its activity for native and non-native pre-tRNA substrates. The oligomerization of HARP creates potential for allosteric communication between the multiple putative substrate binding sites. Therefore, we used single-turnover conditions to try to limit the number of pre-tRNAs associated with each Hth1307 complex. Single-turnover conditions ensure that only a single active site per HARP complex on average will be occupied, therefore minimizing the effects of potential allosteric communication between monomer active sites on reaction kinetics. As with other studies of PRORPs (15, 17, 18, 19, 20, 27, 39, 40, 41, 42), gel-based cleavage assays using 5′-fluorescently labeled pre-tRNAs were used to monitor the percentage of cleaved tRNA over time.

Because structural data indicates that oligomerization is likely required for pre-tRNA binding, we were first interested in determining whether all of the oligomeric states of Hth1307 isolated *via* SEC were catalytically active using three distinct fractions: a monomer, a 14-mer, and a higher-order oligomer. This data shows that the Hth1307 monomeric fraction has no cleavage activity after 2 h compared to the robust activity seen in the Aq880 dodecamer sample and the Hth1307 14-mer and higher-order oligomer peaks (Fig. S10). These results confirm that a higher-order HARP assembly is required for cleavage activity.

In eukaryotic PRORPs, it has been established that increasing the concentration of magnesium within a range may increase the rate of the reaction but also inhibit reactivity above a certain threshold (43). Therefore, it is important to establish the Mg^2+^ dependence in HARP activity because if the nuclease reaction proceeds to completion too rapidly, it is impossible to reliably calculate the observed rate constant. To investigate the effect of the catalytically required Mg^2+^ on nuclease activity, we performed single-turnover experiments to monitor changes in activity upon varying Mg^2+^ concentrations. Using 20 nM *H. thermophilus* pre-tRNA^Lys^ with a 5-nt leader sequence (Ht ptRK^5^, Fig. S11) and 15,000 nM Hth1307 to ensure saturation, reactions were carried out across a range of MgCl_2_ concentrations used to study other PRORP enzymes: 1, 2, 3, and 4.5 mM MgCl_2_ (Fig. S12) (14, 15, 17, 19, 27, 40, 41, 44). The reactions using 1 mM MgCl_2_ enabled us to measure time points that capture the entirety of the reaction without stopped-flow methods. Therefore, we moved forward using 1 mM MgCl_2_ in all subsequent assays.

We were then interested in using single-turnover assays to discover whether Hth1307 showed any specificity for native *versus* non-native pre-tRNAs or had a preference for leader sequence length. Four substrates were selected, shown in Fig. S11. Briefly, *Bacillus subtilis* pre-tRNA^Asp^ with a 5 nt leader sequence and 3′ CCA (Bs ptRD^5 CCA^) has been used extensively in previous characterizations of PRORP enzymes (27, 29, 39, 45, 46), *T. thermophilus* pre-tRNA^Gly^ with a 14-nt leader sequence and no 3′ trailer (Tt ptRG^14^) has been used by other groups investigating HARPs (19), *Saccharomyces cerevisiae* pre-tRNA^Phe^ with a 9-nt leader sequence and no 3′ trailer (Sc ptRF^9^) is a very stable eukaryotic tRNA commonly used in structural and enzymological studies (29, 37), and Ht ptRK is a native substrate from *H. thermophilus* with one of the most prevalent codons in its genome (47). All four of these pre-tRNAs have a 5-nt variable loop and the same number of nucleotides in the acceptor stem, anticodon stem–loop, and T and D loops to limit variability in the general size or folding of the substrates. To investigate whether Hth1307 has a preferred leader sequence length for catalytic activity, we used the native substrate Ht ptRK with 5-, 11-, 15-, and 22-nt leader sequences. A 3′ CCA tail was added to Ht ptRK and Bs ptRD with 5-nt leader sequences to determine if the presence of the 3′ CCA tail impacts the activity of Hth1307. As a control to account for the nucleotides at the cleavage site, the Sc ptRF^9^ substrate used has the same −1 and +1 nucleotides as the native substrate. The purity of the *in vitro* transcribed fluorescently labeled pre-tRNAs was confirmed using denaturing urea-PAGE, shown in Fig. S13.

**Figure 5:**
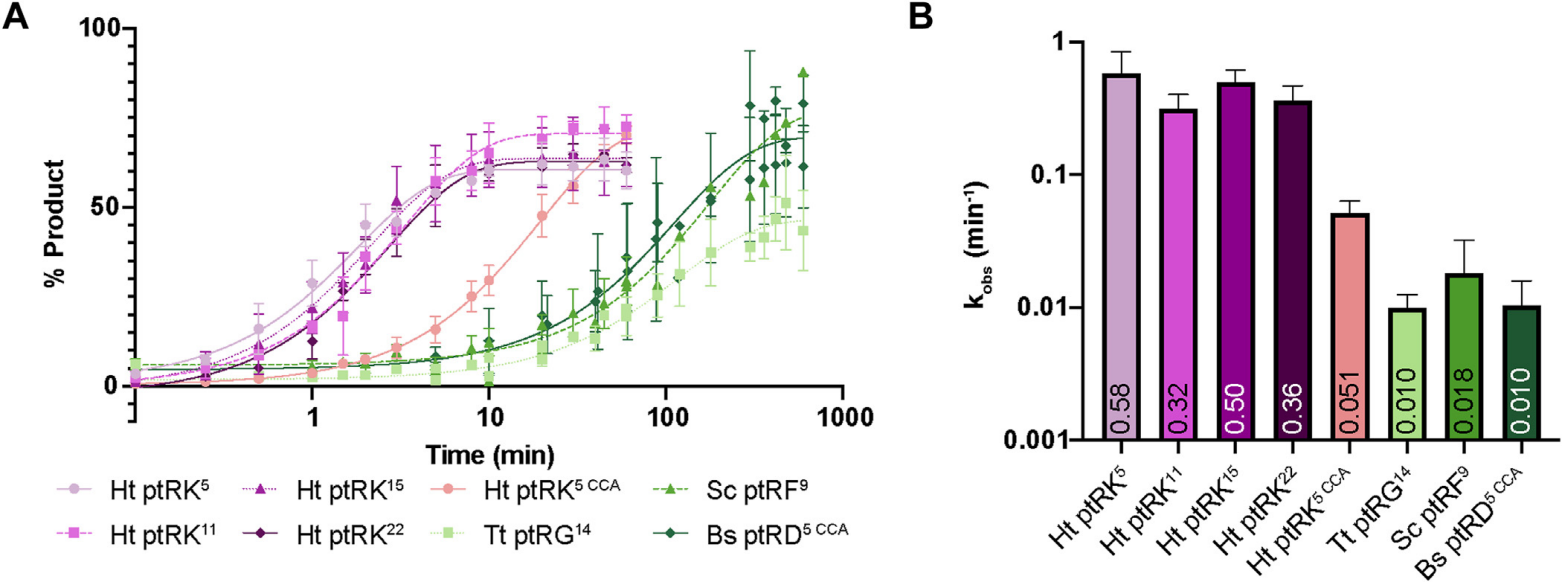
**Single-turnover kinetics assays using Hth1307 and various substrates.***A*, timecourse data of percent product *versus* time for single-turnover reactions carried out with 1000 nM HARP and 2 nM of different pre-tRNA substrates. *B*, comparison of the observed rate constant values for the different substrates tested reveals that Ht HARP has a higher turnover rate with native substrate than other pre-tRNAs commonly used to study RNase enzymes. HARP, Homologs of Aquifex RNase P; Hth1307, *Hydrogenobacter thermophilus* HARP; pre-tRNA, precursor tRNA.

We find that the activity of Ht ptRK did not vary significantly (<2-fold) with differing 5′ leader lengths (5–22 nt) (Fig. 5). However, the presence of a 3′ CCA sequence appears to be an important determinant of substrate selection, as the addition of this sequence slows pre-tRNA cleavage by 10-fold (k_obs,HTpRK5C__C__A_ = 0.05 min^−1^ *versus* k_obs,HTpRK5_ = 0.58 min^−1^). In general, the enzyme inefficiently cleaved non-native pre-tRNAs, exhibiting k_obs_ values ∼60-fold lower than that for native substrates (0.01–0.018 min^−1^). These results demonstrate that Hth1307 exhibits a marked preference for the native substrate.

## Discussion

Here, we present cryo-EM and X-ray crystallography structures of *A. aeolicus* and *H. thermophilus* HARP enzymes. The structures of HARP as a dimer, tetramer, and dodecamer have been reported by different labs and recapitulated here (18, 19, 20). However, our crystal structure of Hth1307 is the first to reveal an even larger oligomer: a 14-mer. This is particularly interesting when considering Supplementary data presented by Teramoto *et al.* that seems to clearly show Hth1307 as a hexamer of dimers in initial cryo-EM 2D classifications (18). It is possible that differences in oligomeric state could be attributed to differences in preparation, such as salt concentrations, the affinity tag used, or buffer pH, which requires further investigation. The overall structure of the 14-mer presented here is a heptamer of dimers, similar to the hexamer of dimers seen in the dodecameric structures previously reported (18, 19, 20). The dimer and tetramer interfaces of the 14-mer are highly similar to the interfaces seen in previously published HARP structures. The ability of Hth1307 to form a 14-mer *in vitro* was confirmed using native MS experiments, providing evidence that a 14-mer structure is not an artifact of our crystallization conditions. Previous publications had established that HARPs can exist as monomers, dimers, tetramers, and dodecamers, but our data indicate that HARPs have a broader range of oligomeric states than previously considered, which may be organism specific. It is unknown what contributes to these varied oligomeric states or their physiological and functional relevance in thermophiles that encode HARP. The propensity for ring-like protein structures in thermophiles and other extremophiles has been previously noted. In *A. aeolicus* specifically, HARP, RNase PH (48), and L-seryl-tRNA^Sec^ selenium transferase (49) all form oligomeric ring structures. It has been proposed that proteins in thermophilic organisms may form oligomeric structures to improve thermostability (50). Because HARP requires at least a tetramer for catalysis (19), which would leave multiple interfaces open to solution, it may be more energetically favorable to form a higher-order oligomer with large interface surface areas.

Comparison of all available HARP structures reveals similar structural features such as the SH domain, a conserved active site, and similar dimer and tetramer interfaces. Briefly, the active sites all contain four aspartate residues and one glutamate residue, which are conserved across HARPs and poised to coordinate two magnesium ions for catalysis, although there is no structural data to confirm this as of yet. All HARP structures also share the same four-helical bundle or coiled-coil formed by two SH domains at the dimer interface. The dimer and tetramer interfaces are primarily formed by hydrophobic contacts, and while the residues have sequence and positional variability, they have conserved function. Despite these similarities, the structures of the larger oligomeric states have significant variation. The previously published Aq880 and Hhal2243 cryo-EM structures form a staggered closing interface with a left-helical turn. Our Aq880 cryo-EM structure also exhibits a staggered closing interface, though to a lesser extent, with our 12-mer only slightly deviating from a symmetrical and perfectly cylindrical arrangement of six dimers. Of significant note is our tetradecameric structure of Hth1307, which is a completely symmetrical and flat cylindrical ring arrangement with no staggered interface. Based on these data, we question whether HARP enzymes form a staggered interface and an overall helical structure under biological conditions. It is unclear if the helical nature of the cryo-EM structures is inherent to the molecule and physiologically relevant or is a result of the air–water interface problem (51, 52, 53). It is also likely that both flat and helically distorted structures are thermodynamically accessible and functionally relevant. For example, helically distorted oligomers may offer clues as to the assembly of HARP oligomers and the expansion by adding, or reduction by expulsion, of a dimer in the staggered interface that allows the complex to adjust their size and oligomeric state.

**Figure 6:**
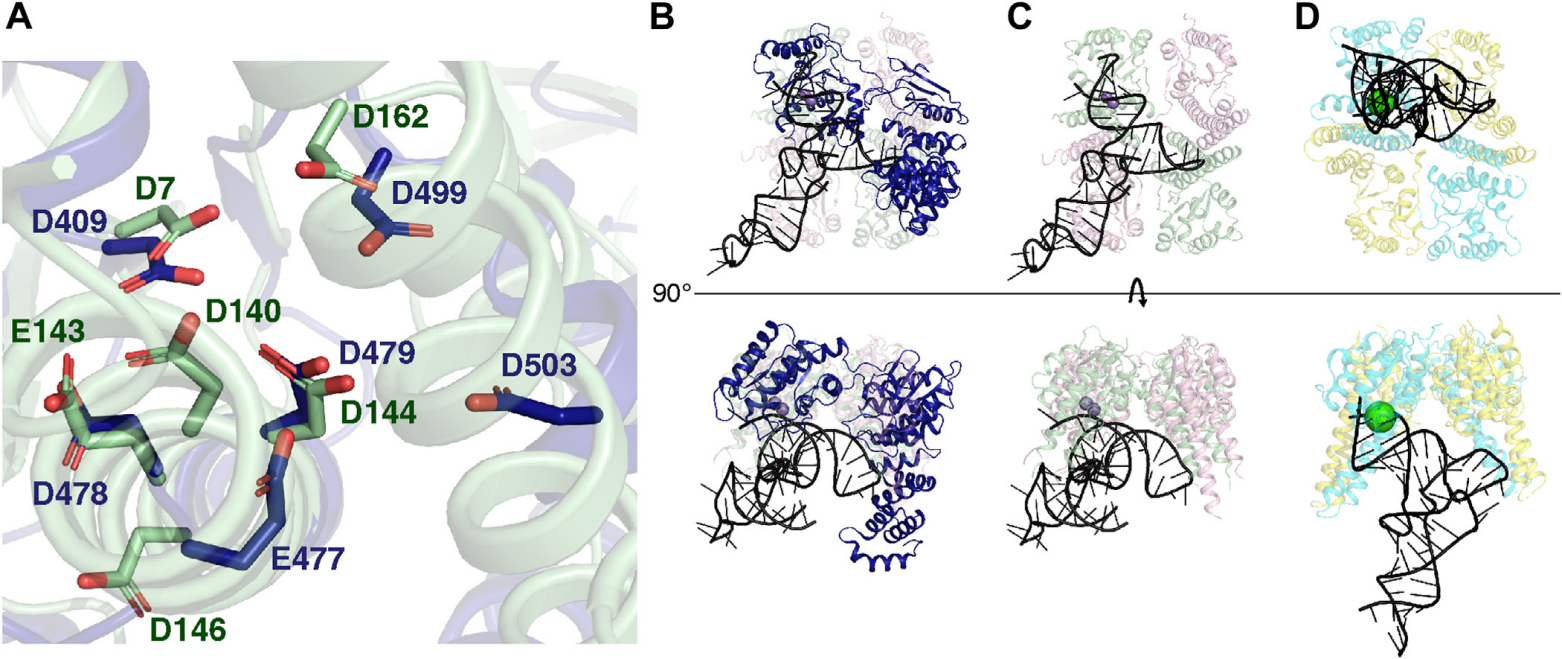
**Alignment of Hth1307 with the MRPP–tRNA**^**Tyr**^**complex using the metallonuclease domain of each provides a new binding model.***A*, active site residues of MRPP (*dark blue*, PDBID: 7ONU) and Hth1307 tetradecamer (*pale green*). *B*, two views of the structural alignment of a tetramer from the Hth1307 tetradecamer (*transparent pink* and *pale green*) to MRPP3 complexed with mt-pre-tRNA^Tyr^. Metal ions were modeled based on manganese ions in the At PRORP1 structure (PDBID: 4G24) and are enlarged 1.5× to showcase the active site. *C*, Hth1307 modeled with mt-pre-tRNA^Tyr^ spanning to interact with diagonally opposite protomers. Metal ions were modeled based on manganese ions in the At PRORP1 structure (PDBID: 4G24) and are enlarged 1.5× to showcase the active site. *D*, Pb HARP (PDBID: 7E8O) is shown in *cyan* and *yellow* and is modeled with *Escherichia coli* pre-tRNA^His^ based on the binding model presented by Li *et al.* (2022). The calcium ion in this structure is enlarged 1.5× to showcase the active site. HARP, Homologs of Aquifex RNase P; Hth1307, *Hydrogenobacter thermophilus* HARP; pre-tRNA, precursor tRNA; PRORP, protein-only RNase P.

In addition to comparing HARP structures, we aligned the active sites of our Hth1307 structures with the active site of the cryo-EM structure of the human mitochondrial PRORP in complex with mitochondrial pre-tRNA^Tyr^ (Protein Data Bank [PDB] ID: 7ONU) to gain insight into how HARPs may bind pre-tRNAs (Fig. 6) (32). Binding is likely to occur across two dimers as indicated by previous work that shows little to no cleavage activity from dimeric Aq880 (19). Accordingly, previously proposed HARP-binding models showed the T and D loops interacting with the SH domain of the protomer directly opposite the active site (Fig. 6*D*). This model has the potential for two substrates binding per tetramer interface, for a total of up to 12 tRNAs binding the 12-mer. However, based on the alignment of our Hth1307 tetramer with the MRPP complex, it appears the active site of Hth1307 HARP is oriented such that the T and D loops of a pre-tRNA substrate would interact with the SH domain of the protomer diagonally opposite the active site (Fig. 6*C*), with a maximum of one substrate binding per tetramer interface, for a total of up to seven tRNAs binding the 14-mer. This could explain why a tetramer is the minimum catalytic unit of HARP enzymes. We do note clashing of the pre-tRNA with HARP in our alignment, specifically between the α6 helix of one protomer and the acceptor stem of the tRNA and between the α6 helix of the diagonally opposite protomer and the T and D loops. This suggests a structural rearrangement of the SH domain is required for our proposed binding model to be correct. When modeling the tRNA interacting with the protomer directly opposing the active site, we still observe clashing. A structure of HARP complexed with a pre-tRNA that inserts into the active site is required to confirm either of the proposed binding models.

In addition to our structural characterization of HARP, we investigated the single-turnover activity of Hth1307 using native and non-native substrates. Our experiments indicate a strong preference for the native Ht ptRK substrate, with activity toward native Ht ptRK^5^ with the 3′ CCA tail being 10-fold lower and activity toward non-native tRNAs being 60-fold lower. The disparity in observed rate constants between the different substrates used here is unlikely to be due to the phosphodiester cleavage and is instead likely due to slow binding of non-native substrates or a protein conformational change associated with substrate binding. Our attempts to characterize the binding affinity of Hth1307 for different substrates using electrophoretic mobility shift assays (data not shown) were complicated by the appearance of multiple bands, indicative of either multiple tRNAs binding to one oligomer or multiple oligomeric states of Hth1307 binding to tRNA. Regardless, the substrate specificity displayed by Hth1307 is uncommon in PRORP enzymes, which are generally considered more promiscuous and have similar activities for native and non-native substrates (15, 27, 41).

It is difficult to draw direct comparisons to previous studies of HARP activity due to the different reaction conditions, such as Mg^2+^ concentration, used in various papers. However, the catalytic mechanism is likely conserved across all types of PRORP enzymes as evidenced by their conserved active sites and similar kinetic activities (Table S3). The Hth1307 single-turnover activity (k_obs_) for native substrates ranged 0.30 to 0.55 min^−1^, which is similar to that of Aq880 with the non-native substrate Tt ptRG^14^ (0.62–2.06 min^−1^), MRPP3 with mitochondrial pre-tRNAs (0.03–0.80 min^−1^), At PRORP1 with non-native substrates (0.05–0.69 min^−1^), and At PRORP2 with non-native substrates (0.03–0.31 min^−1^) (15, 17, 19, 40, 41).

We conclude that HARPs have a broader range of oligomeric states than previously considered, undergo conformational changes to accommodate pre-tRNA substrates, are sensitive to solvent conditions and preferentially recognize native substrates. Based on our structural data, we propose a new binding model for HARPs in which the T and D loops interact with the SH of the diagonally opposing protomer rather than the directly opposing protomer. Further work is required to fully illuminate the physiological role of HARP oligomerization and the effects of HARP conformational changes on tRNA recognition and catalytic activity.

## Experimental procedures

### Expression and purification

The HARP genes from *A. aeolicus* (Aq880) and Hth1307 were synthesized by Integrated DNA Technologies and amplified using PCR. Ligation-independent cloning was used to insert the genes into a pMCSG7 vector (54) that adds an N-terminal His_6_-tag, followed by a tobacco etch virus protease cleavage site. The gene sequence was confirmed using Sanger sequencing through GeneWiz. The resulting plasmid was transformed and expressed in BL21 Rosetta DE3 *E. coli* cells. Cells were grown at 37 °C to an OD_600_ of 0.4 in TB medium and induced by the addition of 0.2 mM IPTG followed by growth for 18 h at 22 °C. Harvested cells were resuspended in Lysis Buffer (20 mM 3-(*N*-morpholino)propanesulfonic acid (MOPS) pH 7.8, 1 M NaCl, 15 mM imidazole, 0.25% Tween-20, 1 mM tris(2-carboxyethyl)phosphine (TCEP)) with the addition of the following per 100 ml: MgCl_2_ to 2.5 mM, 10 mg of lysozyme, 3.5 μl of 250 units/μl benzonase, phenylmethylsulfonyl fluoride to 250 μM, and two cOmplete EDTA-free protease inhibitor cocktail mini-tablets (Roche Applied Science). Cells were lysed using a Q500 sonicator (Qsonica) at 60% amplitude with pulses of 5 s on/10 s off for a total of 10 min on. The lysate was centrifuged for 30 min at 18,000 rpm at 4 °C with a JA-20 rotor (Beckman Coulter). The soluble fraction was applied to a Ni-Sepharose HisTrap HP 5 ml column (Cytiva Life Sciences) pre-equilibrated with Lysis Buffer. The column was washed with five column volumes (CVs) of Lysis Buffer and five CV of nickel wash buffer (20 mM MOPS pH 7.8, 150 mM NaCl, 50 mM imidazole, 1 mM TCEP). Bound proteins were eluted by a gradient from 50 to 500 mM imidazole over 20 CV. Fractions containing HARP were pooled and dialyzed against Ion Exchange Buffer (50 mM Tris pH 8.5, 150 mM NaCl, 1 mM TCEP) overnight at 4 °C. The dialyzed sample was loaded onto a DEAE Sepharose FF column (Cytiva Life Sciences) and eluted with a 150 to 1000 mM NaCl gradient over 20 CV. Fractions containing HARP were pooled and 1 mg tobacco etch virus protease per 50 mg HARP was added to the sample and dialyzed against SEC buffer (50 mM Tris pH 8.5, 150 mM NaCl, 1 mM TCEP) overnight at 4 °C with constant stirring. The sample was then applied to a second Ni-Sepharose column. The flow-through was collected and concentrated with a 10 kDa molecular weight cut-off (MWCO) Amicon Ultra-15 spin column. The sample was then loaded onto a Superdex 200 pg 16/600 (Cytiva Life Sciences) size-exclusion column and eluted with SEC buffer. Peak fractions were pooled and concentrated with a 10 kDa MWCO Amicon Ultra-15 spin column before using in experiments or flash-freezing for storage at −80 °C.

### Crystallization and data collection

Purified protein was concentrated using ultrafiltration (Centricon-30, Amicon) to 10 mg/ml. Crystals of Hth1307 were grown using the sitting drop vapor diffusion method. Crystallization of the purified Hth1307 was initially performed with commercially available screens at 20 °C and 4 °C. Each experiment consisted of mixing 0.5 μl of protein solution (10 mg/ml Hth1307 in 50 mM Tris–HCl pH 8.5, 150 mM NaCl, 1 mM TCEP; protein sample) with 0.5 μl of reservoir solution and equilibrate the drop against 50 μl of reservoir solution in INTELLI-PLATE 96-well trays (Art Robbins). Crystals of the Hth1307 in the tetramer form were grown at 4 °C by mixing protein sample with a crystallization solution consisting of 2 M ammonium sulfate and 0.1 M Bis-Tris pH 5.5. Crystals of the Hth1307 tetramer were briefly transferred to a cryo-protectant solution containing 20% (v/v) glycerol, 1.6 M ammonium sulfate, and 0.08 M Bis-Tris pH 5.5 before harvesting and flash-freezing in liquid nitrogen. Hth1307 crystals in the 14-mer form were grown at 4 °C by mixing protein sample with a crystallization solution containing 1 M sodium malonate pH 5, 0.1 M sodium acetate trihydrate pH 4.5, and 2% (w/v) polyethylene glycol 20,000. Crystals were briefly transferred to a cryo-protectant solution containing 20% (v/v) glycerol, 0.8 M sodium malonate pH 5, 0.08 M sodium acetate trihydrate pH 4.5, and 1.6% (w/v) polyethylene glycol 20,000 before harvesting and flash-freezing in liquid nitrogen.

Diffraction data were collected at the GM/CA 23-ID-B beamline at the Advanced Photon Source, Argonne National Laboratory using a Dectris Eiger X 16 M detector. Data collection and processing statistics are summarized in Table S1. Data for the Hth1307 tetramer were indexed to space group *C*222_1_ (unit cell parameters a = 90.4, b = 108.9, c = 105.7 Å) with two protomers in the asymmetric unit (Matthew’s coefficient V_M_ = 2.92 Å^3^ Da^−1^, 58% solvent content). Data for the Hth1307 14-mer were indexed to space group *P*2_1_22_1_ (unit cell parameters a = 102.3, b = 113.8, c = 155.3 Å) with seven protomers in the asymmetric unit (Matthew’s coefficient V_M_ = 2.88 Å^3^ Da^−1^, 57% solvent content).

### Structure solution of Hth1307 crystallography data

Datasets for the HARP tetramer and tetradecamer crystals were processed using xia2/DIALS (55). Initial phases for the tetramer HARP structure were obtained through molecular replacement with Phaser (56) using a single monomer from *H. halophila* HARP (7OG5 (19)) as a search model. Initial phases for the tetradecameric HARP structure were obtained through molecular replacement with Phaser (56) using a monomer from our tetrameric HARP structure (8SSF). Following molecular replacement, iterative model building and corrections were performed manually using Coot (57). Initial refinement for both structures was conducted using BUSTER version 2.10.4 (58) to rapidly fix Ramachandran, rotamer, and density fit outliers, refining to convergence and adding waters in the final automated round of refinement. Subsequent structure refinements were performed using CCP4 Refmac5 (59) with noncrystallographic symmetry for the tetradecameric structure and without noncrystallographic symmetry for the tetrameric structure, including restrained refinement, coordination minimization, and restrained individual B-factor adjustment with maximum-likelihood targets. PDB-REDO (60) was used to assess the model quality between refinements and to fix any rotamer and density fit outliers automatically. The model quality was evaluated using MolProbity (61). Figures showing crystal structures were generated in PyMOL (62). Structural alignments were performed using the extra_fit function in PyMOL. Angles between tetramers were calculated using anglebetweendomains.py in PyMOL. Atomic coordinates and structure factors for the two crystal structures have been deposited in the PDB.

### Cryo-EM grid preparation, data acquisition, and data processing

Freshly purified Aq880 was screened first using 1% uranyl formate in Morgagni operated at 100 kv and images collected at a magnification of 22,000×. For cryo-EM, 3 μl of Aq880 sample at a concentration of 8 μM was applied to Lacey carbon 200 mesh grid (SPI supplies), which was glow discharged with EasiGlow glow discharge for 60 s (setting 5 mA, 0.26 mBar). The grids were then flash-frozen in liquid ethane using a Thermo Fisher Scientific Vitrobot IV (30 s wait time, 2.5 s blot time, −5 blot force) using Whatman filter paper 1. Cryo-EM images were collected on a Thermo Fisher Scientific Glacios cryo-transmission electron microscope operated at 200 kv using a Gatan K2 Summit camera using Leginon. The image was recorded at a dose rate of 7.7 e^−^/Å^2^ with 200 ms exposure time and collected 40 frames. Defocus ranges ranged from −1.5 to −2.0 μm. Data was processed using cryoSPARC (https://cryosparc.com/), following the workflow of the cryoSPARC software (63). Motion correction (64, 65) and contrast transfer function was performed using full-frame motion correction and path contrast transfer function estimation (65, 66). Particle picking was performed using template picker, picked particles were extracted and performed reference free 2D classification in cryoSPARC (63). After iterative rounds of 2D class average, good classes were used to build two *ab initio* models using *ab initio* reconstruction in cryoSPARC (63). Resulting maps were then used for heterogeneous refinement using the full set of good particles in cryoSPARC. Particles bearing the most homogeneous density were subjected to nonuniform 3D refinement yielding a resolution of 7.8 Å volume. Resolution was estimated according to the Gold-Standard Fourier Shell = 0.143 criterion (67). Figures showing cryo-EM structures were generated using UCSF ChimeraX (68, 69).

### *In vitro* transcription and 5′ end labeling of pre-tRNAs

Pre-tRNA DNA oligos were commercially synthesized by Integrated DNA Technologies and used as a template for *in vitro* transcription reaction. 5′-fluorescent labeling was performed as previously described (14, 40, 42). Reactions were carried out in 30 mM Tris–HCl pH 8, 2 mM spermidine, 0.01% Triton-X, 24 mM MgCl_2_, 5 mM DTT, 4 mM ATP, 4 mM CTP, 4 mM UTP, 0.8 mM GTP, 5 mM guanosine-5′-O-monophosphorothioate, 0.08 U/ml Superase-In, 0.12 U/ml YIPP, 0.28 mM purified T7 RNA polymerase, 0.08 μM DNA template, and 0.08 μM T7 primer. After stopping the reaction by addition of EDTA pH 8 and NaCl to final concentrations of 50 mM and 500 mM, respectively, the samples were washed with degassed 10 mM Tris, 1 mM EDTA (TE) buffer pH 7.2 three times using an Amicon Ultra-4 10 kDa MWCO spin column. The washed pre-tRNA (∼200 μl) was incubated with 20 μl of 45 mM 5-iodoacetamidofluorescein overnight at 37 °C to label the 5′ end. The reaction was stopped by addition of an equal volume of 2× Loading Dye (0.05% Xylene Cyanol Dye, 7 M urea and 0.1 M EDTA pH 8) and run on a 12% urea-polyacrylamide gel. The pre-tRNA was eluted into crush-soak buffer (TE buffer pH 8, 0.1% SDS, and 0.5 M NaCl) overnight at 4 °C. The next day, the mixture was filtered, then concentrated and washed with degassed TE buffer pH 8 using an Amicon Ultra-15 10 kDa MWCO spin column. The pre-tRNA was ethanol precipitated, and the resulting pellet was resuspended in TE buffer pH 8.0. Unlabeled pre-tRNAs were made similarly to labeled ones, except the transcription reaction contained 4 mM GTP and excluded guanosine-5′-O-monophosphorothioate, in addition to omitting the 5′ end labeling step with fluorescein. The concentrations of total and labeled pre-tRNA were measured from absorbance using a Nanodrop spectrophotometer. The concentrations of total and labeled pre-tRNA were measured with absorbance using a Nanodrop (Thermo Fisher Scientific) spectrophotometer and calculated using the extinction coefficients calculated by IDT OligoAnalyzer Tool.

### Single-turnover cleavage assays

Fluorescently labeled pre-tRNA substrates were refolded immediately before each 5′ pre-tRNA cleavage reaction. First, pre-tRNAs were heated at 95 °C for 3 min and cooled down to 37 °C for 15 min. This was followed by the addition of cleavage reaction buffer (30 mM MOPS pH 7.8, 150 mM NaCl, varied MgCl_2_, 1 mM TCEP) and incubation for 15 min at 37 °C.

All single-turnover reactions were performed in the Cleavage Reaction Buffer at 37 °C. To initiate a reaction, a mixture of 1000 nM HARP enzyme in Cleavage Reaction Buffer was added to the 2 nM folded, fluorescently labeled pre-tRNA. For gel-based assays, reaction time points were quenched with equal volumes of 2× Loading Dye and resolved on 20% denaturing urea-polyacrylamide gels. The gels were scanned with an Amersham Typhoon imager, and results were quantified using ImageQuant TL version 10.1 (https://www.cytivalifesciences.com/en/us/shop/protein-analysis/molecular-imaging-for-proteins/imaging-software/imagequant-tl-10-2-analysis-software-p-28619). Data from at least three independent experiments were analyzed using Prism 9 (https://www.graphpad.com/). Equation 1 was used to fit the data to calculate the observed rate constant (*k*) and the standard error from the fitting, where *A* is the endpoint, *B* is the amplitude, and *t* is the time.

(1) $$Fraction\;cleaved=A-Be^{-k_{obs}t}$$

### Analytical SEC to determine concentration-dependent oligomerization

Samples of Hth1307 were diluted in SEC buffer to concentrations ranging from 93.6 nM to 144 μM and filtered with 0.22 μm Costar Spin-X centrifuge tube filters. Samples of 150 μl were loaded onto a Superdex 200 Increase 5/150 GL analytical size-exclusion column (Cytiva Life Sciences) and eluted at 0.17 ml/min, while monitoring the absorbance at 280 nm. Elution profiles were overlaid using Prism 9.

### Native MS of Hth1307

Native mass spectra were collected on a Thermo Fisher Scientific Q-Exactive Ultra-High Mass Range Hybrid Quadrupole-Orbitrap Mass Spectrometer in a positive ionization polarity. Samples were buffer exchanged into 200 mM ammonium acetate pH 7 using Micro Bio-Spin P-6 gel columns and directly infused *via* nanoelectrospray ionization. Nanoelectrospray ionization was performed using borosilicate needles pulled and coated in-house with a Sutter p-97 Needle Puller and a Quorum SCX7620 mini sputter coater, respectively. MS parameters of note include a capillary temperature between 200 to 250 °C, the high-collisional dissociation cell set between 10 to 20 V, and the spray voltage set between 1.0 to 1.4 kV. Detector optimization and transfer optics were set to high *m/z* and spectra were collected at 1 min. The acquired native mass spectra were deconvoluted using UniDec (70).

## Data availability

Structural data are deposited in the RCSB PDB. PDB-ID codes: 8SSF and 8SSG.

## Supporting information

This article contains supporting information (14, 15, 17, 19, 27, 40, 41, 44).

## Conflict of interest

The authors declare that they have no conflicts of interest with the contents of this article.
