# Supplementary material for: Bacterial RNA-free RNase P: Structural and functional characterization of multiple oligomeric forms of a minimal protein-only ribonuclease P
Source: J Biol Chem. 2023 Oct 6;299(11):105327. doi: 10.1016/j.jbc.2023.105327 (PMC10652100; doi:10.1016/j.jbc.2023.105327)
Supplement: Supporting Figures S1–S13 and Tables S1–S3 [file mmc1.docx]

**SUPPORTING INFORMATION**

**Bacterial RNA-free RNase P: structural and functional characterization of multiple oligomeric forms of a minimal protein-only ribonuclease P**

**Catherine A. Wilhelm^1^, Leena Mallik^1,2^, Abigail L. Kelly^1^, Shayna Brotzman^1^, Johnny Mendoza^1^, Anna G. Anders^1^, Suada Leskaj^1^, Carmen Castillo^3^, Brandon T. Ruotolo^1^, Michael A. Cianfrocco^3^, Markos Koutmos^1,3,4^***

^1^ Department of Chemistry, University of Michigan, Ann Arbor, MI, 48109

^2^ Center for Computational and Genomic Medicine and Department of Pathology and Laboratory Medicine, Children's Hospital of Philadelphia, Philadelphia, PA, USA. 19104

^3^ Department of Biological Chemistry, University of Michigan, Ann Arbor, MI, 48109

^4^ Program in Biophysics, University of Michigan, Ann Arbor, MI, 48109

* to whom correspondence should be addressed: Markos Koutmos, [mkoutmos@umich.edu](mailto:mkoutmos@umich.edu).

**TABLE OF CONTENTS**

| **Supplemental Figure 1.** Cryo-EM data processing. | 2 |
| --- | --- |
| **Supplemental Figure 2.** Chromatograms from purifications and standards. | 4 |
| **Supplemental Figure 3.** Detailed native MS data. | 5 |
| **Supplemental Figure 4.** Chromatograms of Hth1307 showing A280 and A260. | 6 |
| **Table S1.** Crystallography statistics. | 8 |
| **Supplemental Figure 5.** Details of the Hth1307 structure and interfaces. | 9 |
| **Supplemental Figure 6.** Alignment of sequences of structurally characterized HARPs. | 10 |
| **Table S2.** Interface data determined by PISA. | 11 |
| **Supplemental Figure 7.** Hth1307 surface potential map. | 12 |
| **Supplemental Figure 8.** Alignment of unique HARP monomers. | 13 |
| **Supplemental Figure 9.** Alignment of available 12-mer structures and the 14-mer. | 14 |
| **Supplemental Figure 10.** Gel-based assays to confirm activity of Aq880 and Hth1307. | 15 |
| **Supplemental Figure 11.** Illustrated sequences of pre-tRNA substrates. | 16 |
| **Supplemental Figure 12.** Cleavage reaction time courses with varied [Mg]. | 17 |
| **Supplemental Figure 13.** Urea-PAGE showing pre-tRNA purity. | 18 |
| **Table S3.** Comparison of PRORP kinetics data from a variety of articles. | 19 |


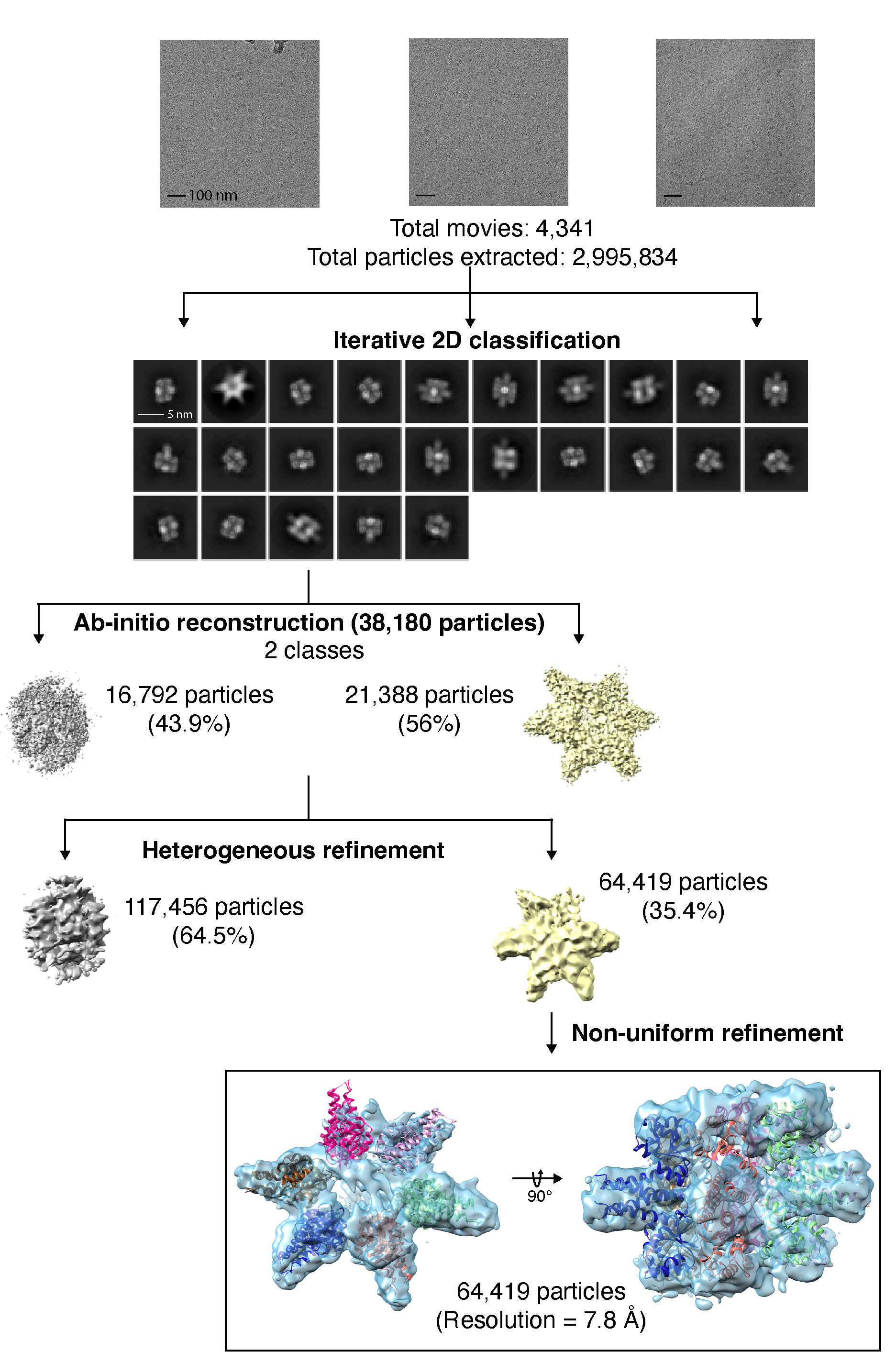


**Supplemental Figure 1.** Cryo-EM data processing workflow. Cryo-EM micrographs were collected with a Glacios microscope operating at 200 kV and a pixel size of 1.25 Å. Computational processing was performed using cryoSPARC v3.1. The black scale bar on the micrographs is 100 nm. The white scale bar on the class averages is 5 nm. Full details of data collection and processing can be found in the Methods section.

**Supplemental Figure 2.** **A-B.** Representative elution profiles from the size exclusion chromatography with the HiLoad 16/600 Superdex 200 pg column for both Aq880 and Hth1307, showing that Hth1307 has a sub-population that elutes at a higher molecular weight. The small peak at 40 mL in **A.** is the elution of aggregated protein after the dead volume of the column. **C.** Elution profile of Bio Rad Gel Filtration Standards showing the absorbance at 280 nm on the Superdex 200 Increase 5/150 GL column used for analytical size exclusion chromatography. **D.** Standard curve from the elution profile on the left. This curve was used to approximate the molecular weight, and therefore oligomeric state, of Hth1307 at varying concentrations. Based on the average elution volume of 1.54 mL, the molecular weight oof the protein eluting from the column is calculated to be 399.762 kDa, or approximately an 18-mer. However, larger molecular weights have more error in such standard curves due to the logarithmic relationship between elution volume and molecular weight. The oligomeric state of Hth1307 cannot be accurately determined by size exclusion chromatography but does not appear to change elution volume, and therefore oligomeric state, based on concentration.


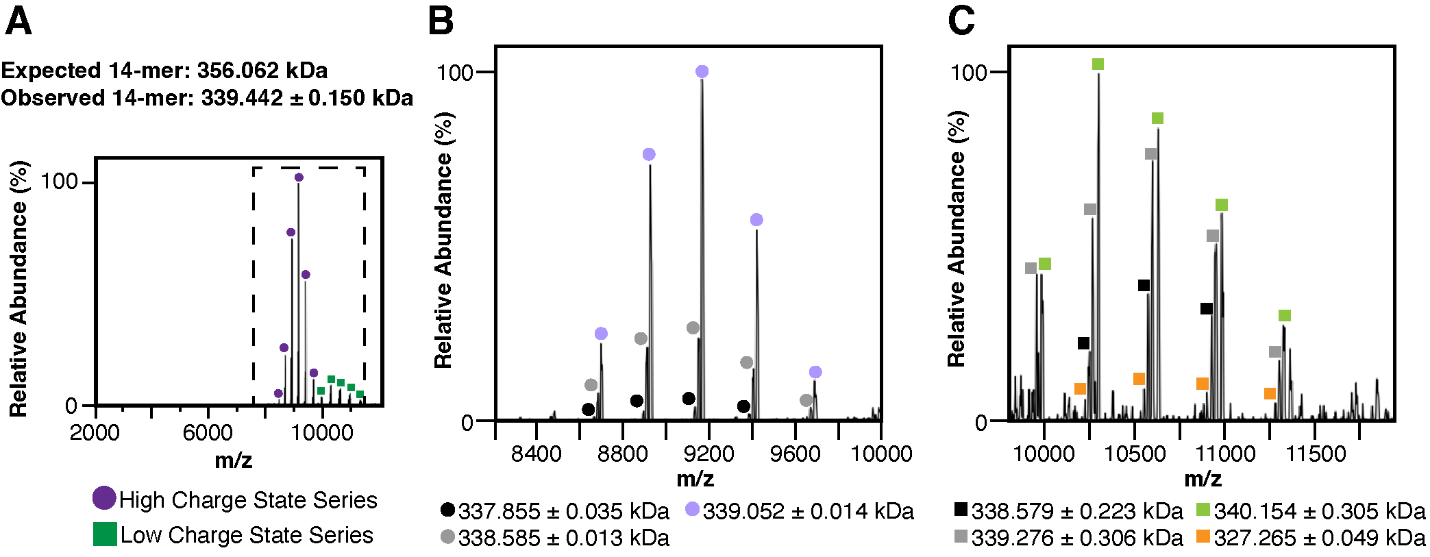


**Supplemental Figure 3.** Detailed analysis of native mass spectra results for Hth1307. **A.** Native mass spectra of His-tagged Hth1307. Upon a closer look at the **B.** high and **C.** low charge state series, additional masses are observed. In each of these series, masses differing by between 200-600 Da are observed and could be related to cleavage of a portion of the labile His-tag or loss of the magnesium ions from the active site. **C.** Within the low charge state series, a new series is observed (orange squares) and relates to a 14-mer with the loss of 9-10 His-tags.

**Supplemental Figure 4.** Elution profiles of Hth1307 injected onto a Superdex 200 Increase 5/150 GL column at concentrations ranging from 250 nM to 12 μM, showcasing the ratio of the absorbance at 280 and 260 nm, corresponding to protein and RNA respectively. Lower sample concentrations did not yield reliable A_260_ measurements.

**Table S1.** Data collection and refinement statistics for x-ray crystallography structure determination of *Hydrogenobacter thermophilus* HARP (Hth1307). Values in parentheses are for the highest resolution shell.

|  | Hth1307 tetramer | Hth1307 tetradecamer |
| --- | --- | --- |
| **Data collection** |  |  |
| Space group | C222_1_ | P2_1_22_1_ |
| Cell dimensions |  |  |
| *a*, *b*, *c* (Å) | 90.5, 109.2, 106.1 | 102.3, 113.7, 155.3 |
| α, β, γ (°) | 90, 90, 90 | 90, 90, 90 |
| Resolution (Å) | 69.72 (2.50) | 29.86 (3.20) |
| *R*_sym_ or *R*_merge_ | 30 (396) | 12 (196) |
| *I* / σ*I* | 6.1 (1.3) | 12.1 (1.2) |
| Completeness (%) | 100 (100) | 100 (99.8) |
| Redundancy | 8.5 (8.6) | 6.8 (7.2) |
|  |  |  |
| **Refinement** |  |  |
| Resolution (Å) | 48.45(2.60) | 29.74 (3.20) |
| No. reflections | 18585/2076 | 30570/1542 |
| *R*_work_ / *R*_free_ | 21.1/25.4 | 16.2/23.7 |
| No. atoms |  |  |
| Protein | 2877 | 19967 |
| Ligand/ion | 15 |  |
| Water | 12 | - |
| *B*-factors |  |  |
| Protein | 76.25 | 116.4 |
| Ligand/ion | 99 |  |
| Water | 63 |  |
| R.m.s. deviations |  |  |
| Bond lengths (Å) | 0.010 | 0.013 |
| Bond angles (°) | 2.103 | 1.890 |


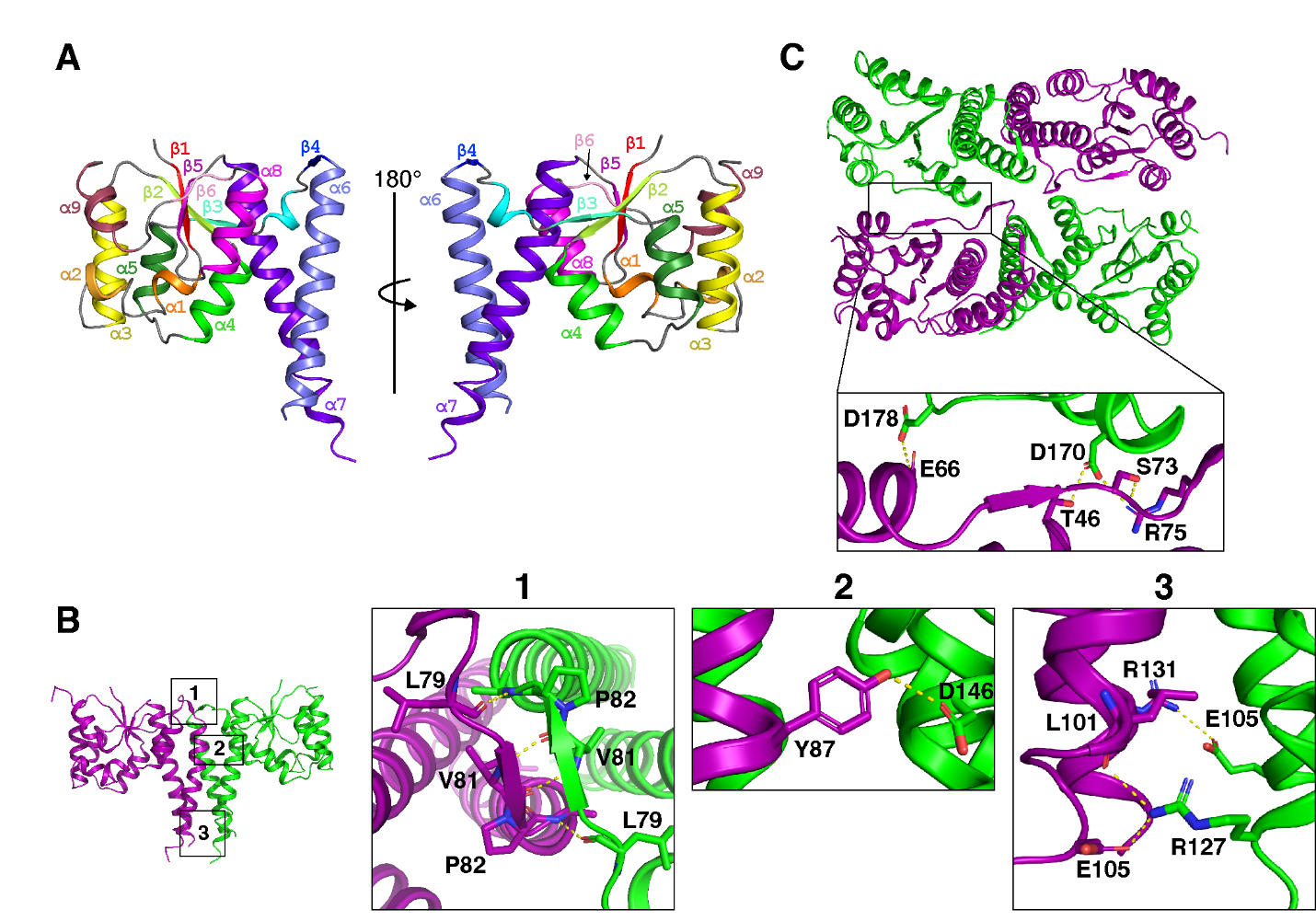


**Supplemental Figure 5.** Details of the Hth1307 structure and interfaces **A.** An Hth1307 monomer with α helices and β strands numbered and color-coded. **B-C.** Dimer and tetramer interfaces in the Hth1307 tetramer. The residues involved in polar contacts are labeled with dashed yellow lines indicating hydrogen bonding and salt bridges. **B.** The dimer interface is a 4 helical bundle formed by the spike helix domains of both dimers. In addition to several hydrophobic interactions, the interface is stabilized by polar contacts in three areas. **C.** The tetramer interface forms between the metallonuclease domains of the monomers rather than the spike helix domains.


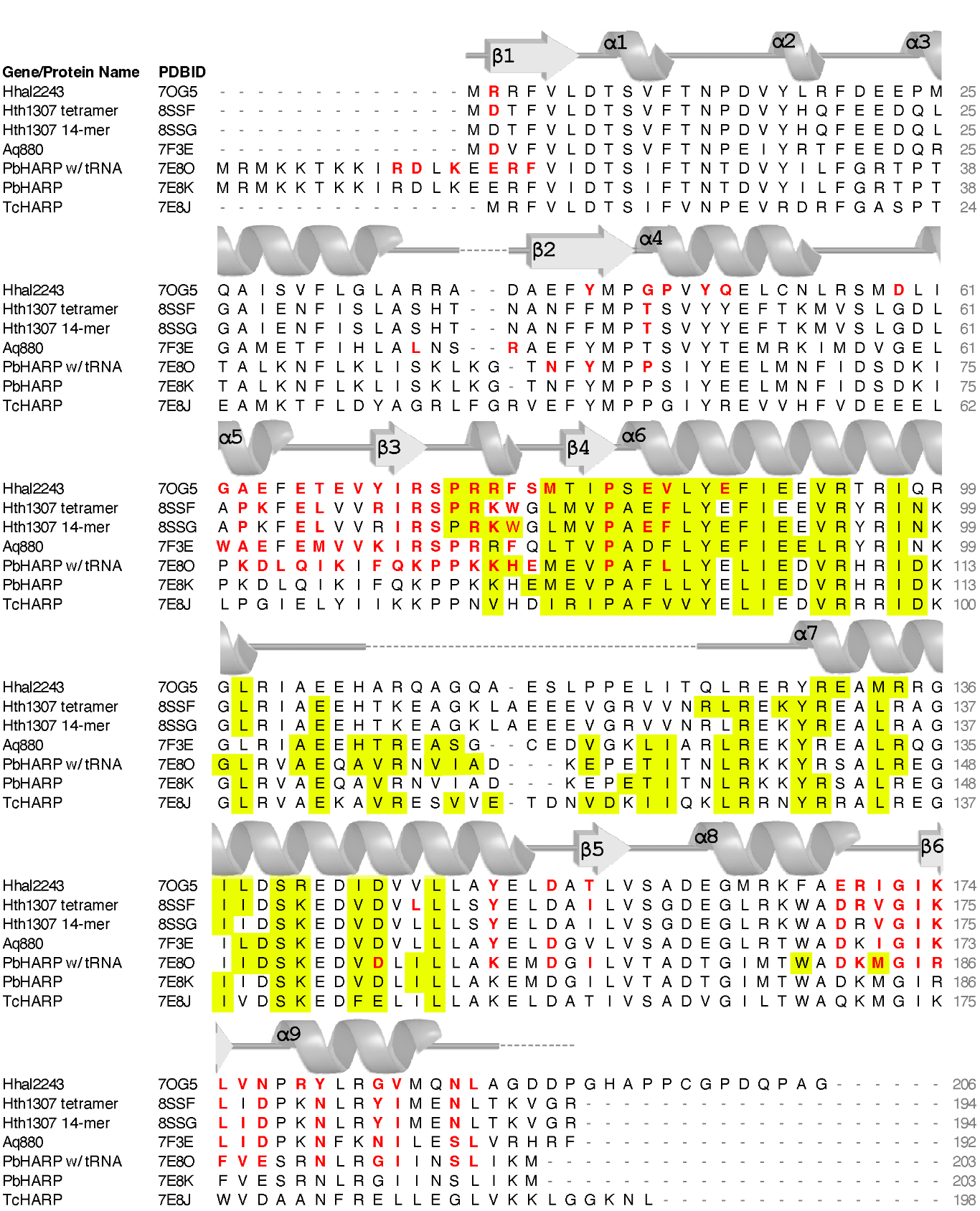


**Supplemental Figure 6.** Alignment of sequences of structurally characterized HARPs was performed using Clustal Omega. The secondary structure diagram above the alignments corresponds to the Hth1307 tetramer structure and was created using PISA. Residues highlighted in yellow or shown in red are part of the dimer and tetramer interface respectively.

**Table S2.** Interface data determined by PISA for both Hth1307 structures presented in this paper to demonstrate differences in the surface area and free energy.

| **Hth1307 14mer (8SSG)** | | | | | | | | | | | | | | | |  |
| --- | --- | --- | --- | --- | --- | --- | --- | --- | --- | --- | --- | --- | --- | --- | --- | --- |
| **Interface** | **Chain 1** | **^i^N_at_** | **^i^N_res_** | **Surface, A^2^** | **Chain 2** | **^i^N_at_** | **^i^N_res_** | **Surface, A^2^** | **Interface area, A^2^** | **Δ^i^G (kcal/mol)** | **Δ^i^G p-value** | **N_HB_** | **N_SB_** | **N_DS_** | **CSS** |  |
| **Dimer** | B | 130 | 34 | 10244 | A | 127 | 32 | 9993 | 898.2 | -12.7 | 0.026 | 10 | 8 | 0 | 1 |  |
|  | D | 157 | 39 | 10100 | C | 148 | 40 | 10868 | 1074 | -12.2 | 0.06 | 15 | 11 | 0 | 1 |  |
|  | F | 135 | 33 | 10449 | E | 141 | 36 | 10017 | 939.6 | -15 | 0.012 | 8 | 8 | 0 | 1 |  |
|  | H | 154 | 37 | 10567 | G | 153 | 37 | 10573 | 1121.9 | -14.2 | 0.023 | 8 | 12 | 0 | 1 |  |
|  | J | 138 | 36 | 10021 | I | 136 | 33 | 10446 | 941.1 | -15.1 | 0.011 | 8 | 8 | 0 | 1 |  |
|  | L | 147 | 40 | 10872 | K | 157 | 39 | 10100 | 1074.1 | -12.3 | 0.059 | 15 | 11 | 0 | 1 |  |
|  | N | 128 | 32 | 10003 | M | 129 | 34 | 10241 | 897.1 | -12.6 | 0.026 | 10 | 8 | 0 | 1 |  |
|  | Average: | | | | | | | | 992.3 | -13.4 | 0.031 | 10.6 | 9.4 | 0 | 1 |  |
| **Tetramer** | C | 60 | 12 | 10868 | A | 83 | 24 | 9993 | 504.8 | -2.7 | 0.558 | 4 | 0 | 0 | 0 |  |
|  | D | 79 | 25 | 10100 | B | 61 | 13 | 10244 | 519.7 | -4.9 | 0.327 | 6 | 3 | 0 | 0 |  |
|  | E | 64 | 13 | 10017 | C | 81 | 22 | 10868 | 510.2 | -2.9 | 0.463 | 4 | 0 | 0 | 0 |  |
|  | F | 79 | 23 | 10449 | D | 63 | 13 | 10100 | 487.1 | -3.9 | 0.407 | 5 | 0 | 0 | 0 |  |
|  | G | 60 | 12 | 10573 | E | 83 | 23 | 10017 | 507.9 | -5.5 | 0.271 | 3 | 2 | 0 | 0 |  |
|  | H | 80 | 23 | 10567 | F | 65 | 12 | 10449 | 492.6 | -3.4 | 0.46 | 6 | 0 | 0 | 0 |  |
|  | I | 63 | 12 | 10446 | G | 80 | 24 | 10573 | 494.1 | -3.4 | 0.452 | 6 | 0 | 0 | 0 |  |
|  | J | 81 | 23 | 10021 | H | 60 | 12 | 10567 | 508.6 | -5.4 | 0.271 | 3 | 2 | 0 | 0 |  |
|  | K | 62 | 13 | 10100 | I | 80 | 23 | 10446 | 486.8 | -3.8 | 0.41 | 5 | 0 | 0 | 0 |  |
|  | L | 84 | 22 | 10872 | J | 64 | 13 | 10021 | 510.7 | -2.9 | 0.469 | 4 | 0 | 0 | 0 |  |
|  | M | 61 | 13 | 10241 | K | 79 | 25 | 10100 | 520.8 | -4.9 | 0.328 | 6 | 3 | 0 | 0 |  |
|  | M | 76 | 22 | 10241 | A | 61 | 11 | 9993 | 482.6 | -4.9 | 0.375 | 4 | 0 | 0 | 0 |  |
|  | N | 83 | 24 | 10003 | L | 60 | 12 | 10872 | 505.7 | -2.7 | 0.559 | 4 | 0 | 0 | 0 |  |
|  | N | 60 | 11 | 10003 | B | 76 | 22 | 10244 | 484.1 | -4.9 | 0.366 | 4 | 0 | 0 | 0 |  |
|  | Average: | | | | | | | | 501.1 | -4.0 | 0.408 | 4.6 | 0.7 | 0 | 0 |  |
|  | | | | | | | | | | | | | | | |  |
| **Hth1307 tetramer (8SSF)** | | | | | | | | | | | | | | | |  |
| **Interface** | **Chain 1** | **^i^N_at_** | **^i^N_res_** | **Surface, A^2^** | **Chain 2** | **^i^N_at_** | **^i^N_res_** | **Surface, A^2^** | **Interface area, A^2^** | **Δ^i^G (kcal/mol)** | **Δ^i^G p-value** | **N_HB_** | **N_SB_** | **N_DS_** | **CSS** |  |
| **Dimer** | B | 150 | 36 | 10003 | A | 139 | 34 | 9981 | 1459.3 | -27.1 | 0.003 | 5 | 3 | 0 | 1 |  |
| **Tetramer** | B | 61 | 11 | 10003 | A | 75 | 21 | 9981 | 589.5 | -6.9 | 0.192 | 0 | 2 | 0 | 0.02 |  |
|  | | | | | | | | | | | | | | | |  |
| ^i^N_at_ | | the number of interfacing atoms in the corresponding structure | | | | | | | | | | | | | |  |
| ^i^N_res_ | | the number of interfacing residues in the corresponding structure. | | | | | | | | | | | | | |  |
| Surface, A^2^ | | the total solvent accessible surface area in square Ångstroms | | | | | | | | | | | | | |  |
| Interface area, A^2^ | | calculated as difference in total accessible surface areas of isolated and interfacing structures divided by two. | | | | | | | | | | | | | |  |
| Δ^i^G (kcal/mol) | | the solvation free energy gain upon formation of the interface | | | | | | | | | | | | | |  |
| Δ^i^G p-value | | the P-value of the observed solvation free energy gain | | | | | | | | | | | | | |  |
| N_HB_ | | the number of potential hydrogen bonds across the interface | | | | | | | | | | | | | |  |
| N_SB_ | | the number of potential salt bridges across the interface | | | | | | | | | | | | | |  |
| N_DS_ | | the number of potential disulfide bonds across the interface | | | | | | | | | | | | | |  |
| CSS | | the Complexation Significance Score, which indicates how significant for assembly formation the interface is | | | | | | | | | | | | | |  |


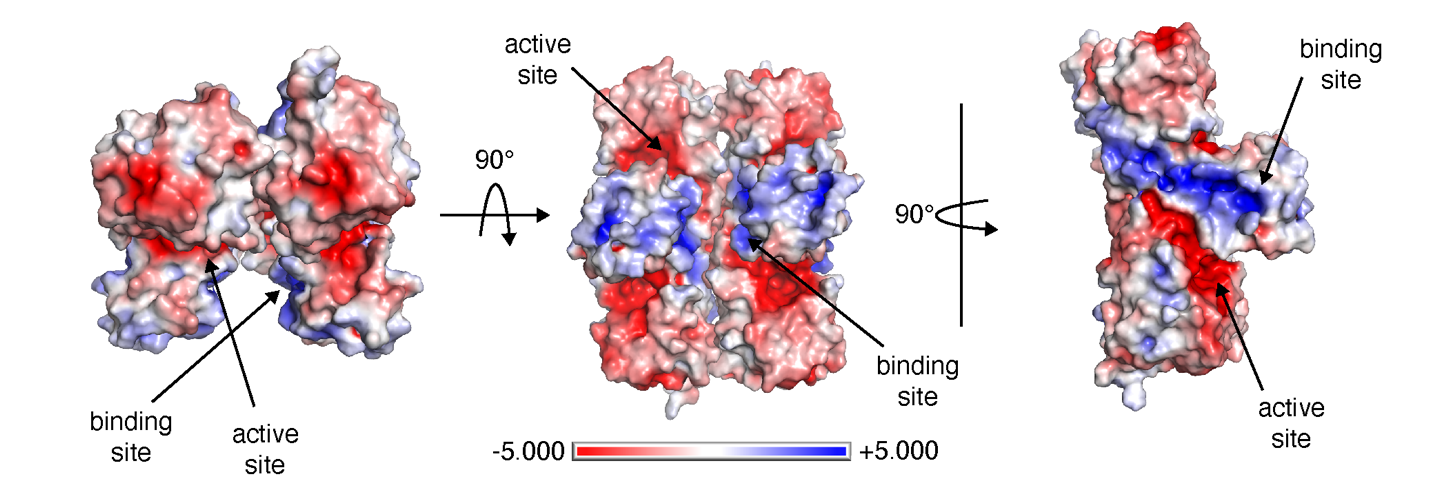


**Supplemental Figure 7.** Hth1307 surface shown with potential isocontours, calculated using the APBS Electrostatics plugin for PyMOL, with a range of +5 kT/e (blue) to -5 kT/e (red).


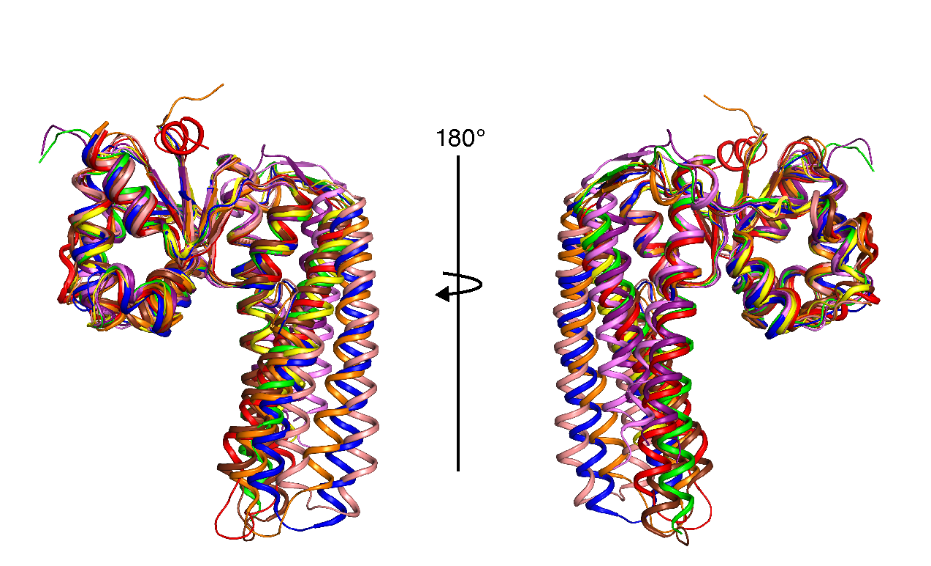


**Supplemental Figure 8.** Monomers from Hth1307, Aq880, Hhal2243, Tc HARP, and Pb HARP aligned by the metallonuclease domain. Monomers in violet, deep purple, and green are Hth1307. Yellow is Hhal2243 (PDBID: 7OG5). Blue is Aq880 (PDBID: 7F3E). Brown is Tc HARP (PDBID: 7E8J). Red is Pb HARP without tRNA bound (PDBID: 7E8K). Orange and salmon are Pb HARP with tRNA bound (PDBID: 7E8O).


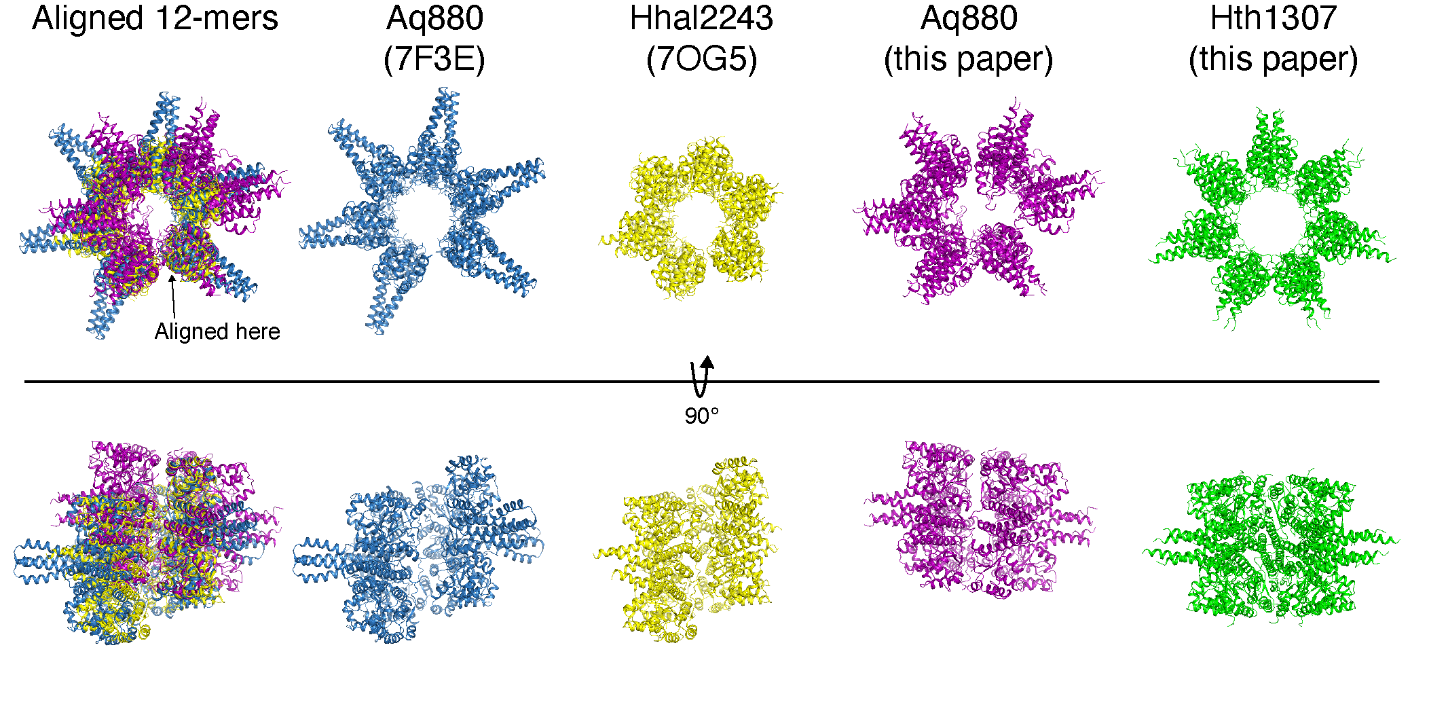


**Supplemental Figure 9.** Alignment of the three available dodecameric structures of HARP next to the Hth1307 tetradecamer from two angles shows the differences in the helical turn and closing interface of the four structures.


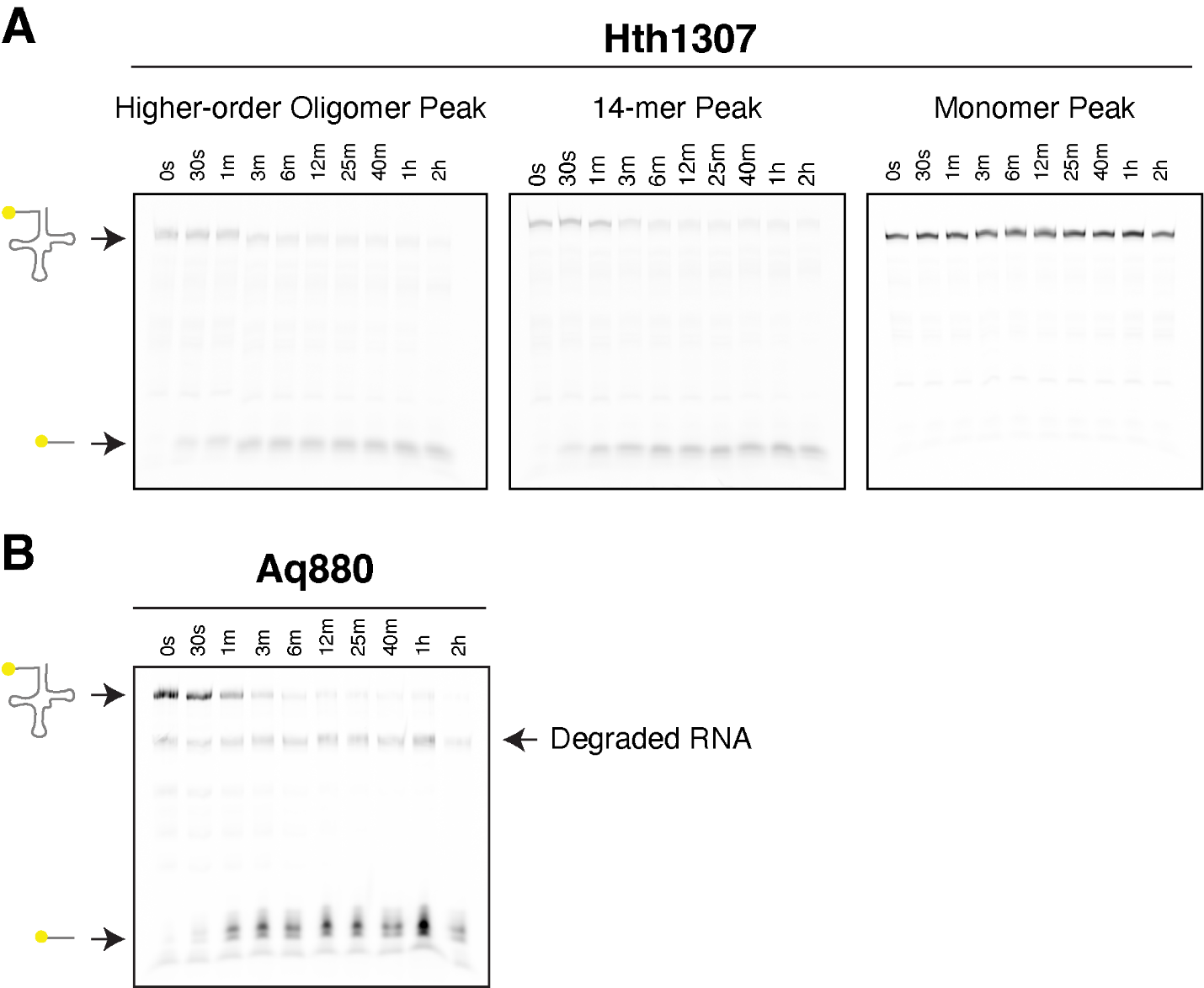


**Supplemental Figure 10.** Enzymatic activity of purified HARP proteins was confirmed via gel-based multiple turnover assays using **A.** 50 nM Hth1307 from different SEC peaks or **B.** 50 nM Aq880 and 5 μM Bs ptRD^5^ at 5 mM MgCl_2_ and 300 mM NaCl. The Hth1307 monomer has no activity compared to the higher order oligomers.


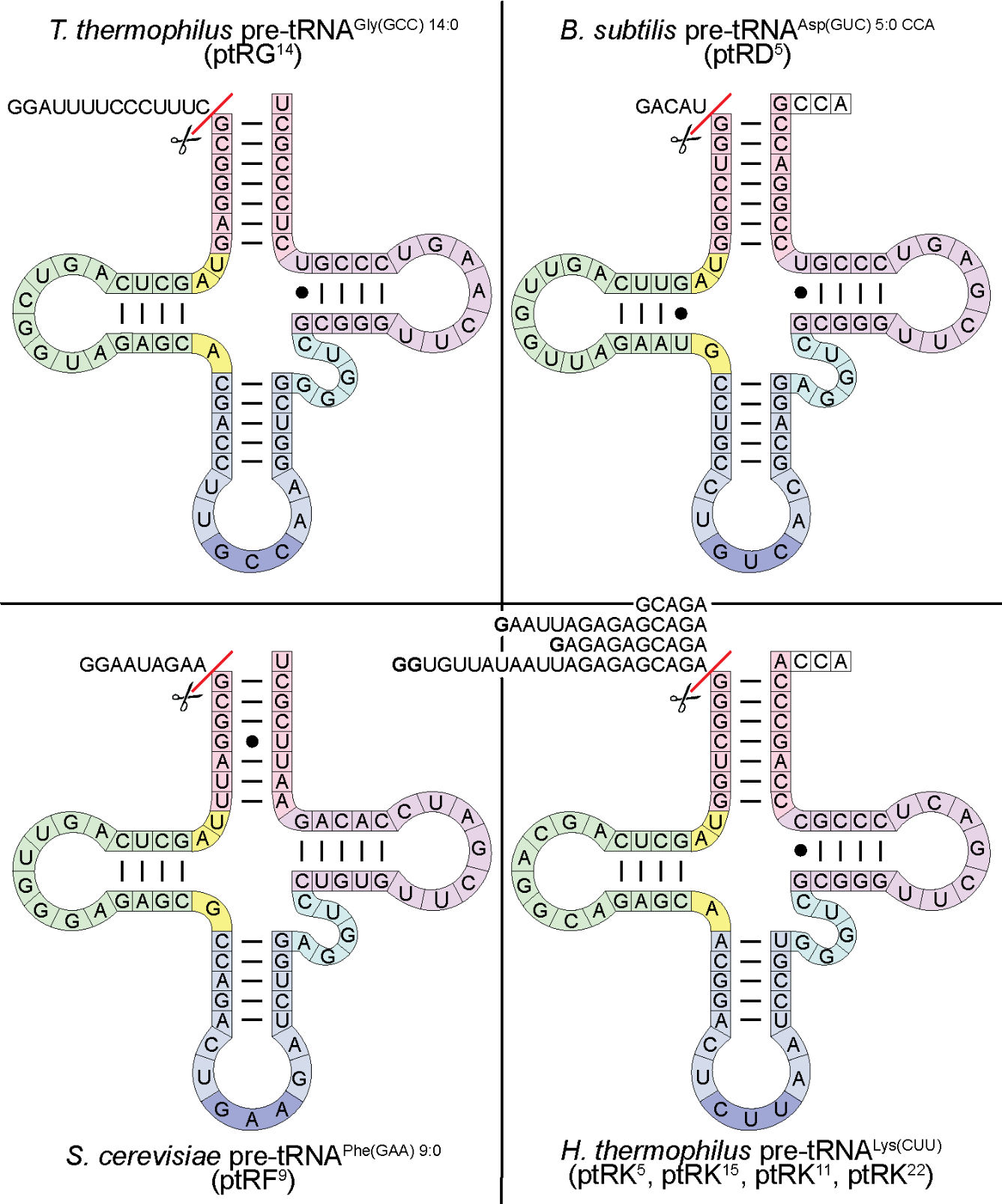


**Supplemental Figure 11.** Illustrated sequences of the four pre-tRNA substrates selected for the single turnover assays.

**Supplemental Figure 12.** The formation of product over time at different magnesium concentrations was monitored using 20.75 μM Hth1307 and 20 nM fluorescently labeled Sc ptRF^9^.


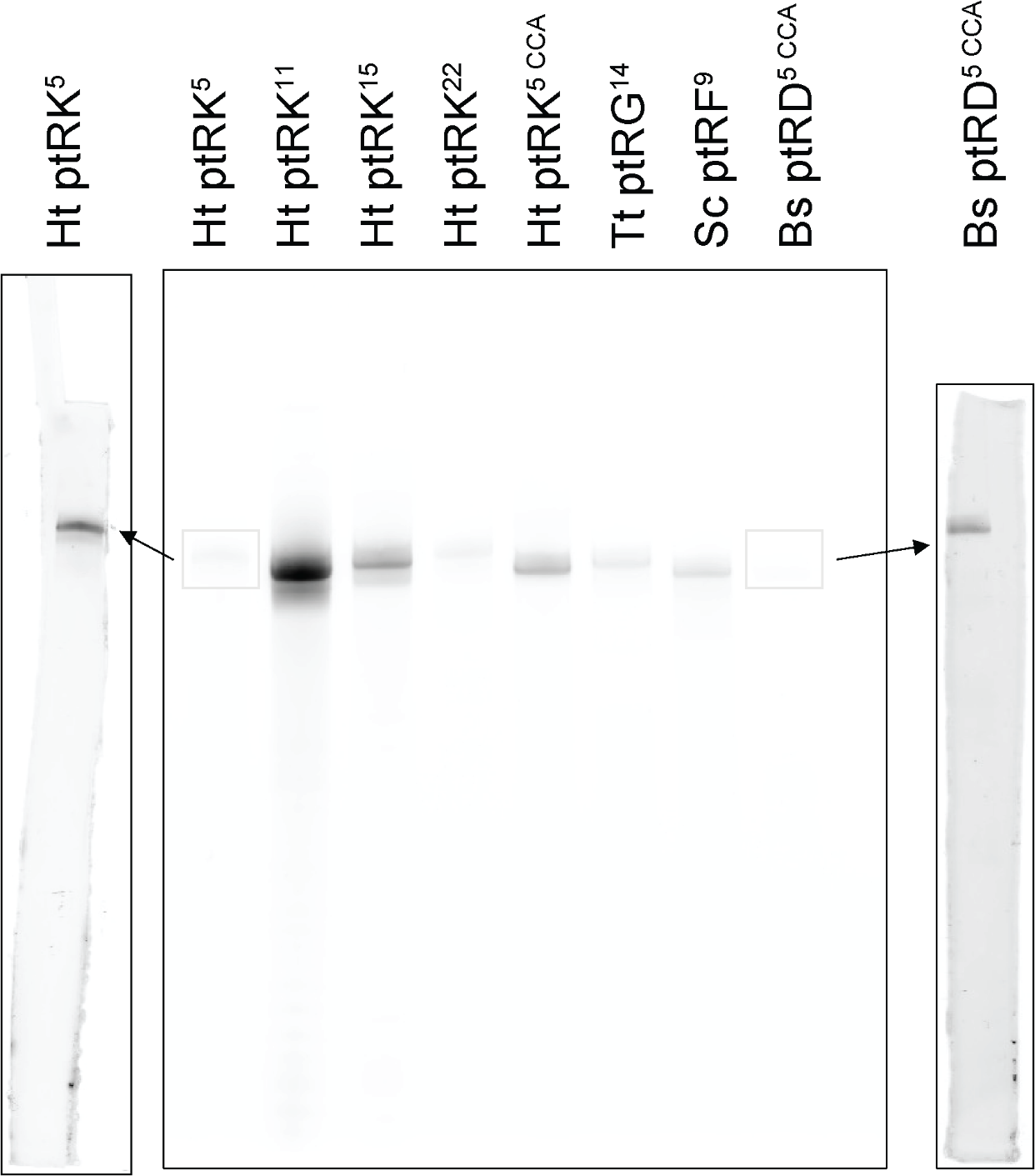


**Supplemental Figure 13.** The quality of the pre-tRNAs used in our kinetic assays was surveyed using denaturing urea-PAGE. Due to the low labeling efficiency of Ht ptRK^5^ and Bs ptRD^5 CCA^, boxed in light grey, those lanes were excised from the gel and imaged with higher amplification of the signal.

**Table S3.** Comparison of PRORP kinetics data from a variety of articles. (Pre-tRNA names are abbreviated by the following naming convention: Bs ptRD5:1 is *Bacillus subtilis* pre-tRNA^Asp^ with a 5-nt leader sequence and a 1-nt trailer sequence. Abbreviations used: Ht, *Hydrogenobacter thermophilus*; Tt, *Thermus thermophilus*; Sc, *Saccharomyces cerevisiae*; Bs, *Bacillus subtilis*; At, *Arabidopsis thaliana*; mito, mitochondria; chloro, chloroplast; nuc, nuclear; Hs, *Homo sapiens.*)

| **Protein** | **[Protein]** | **Pre-tRNA Substrate** | **[RNA]** | **Native substrate?** | **[Mg]** | **[NaCl]** | **Temp (°C)** | **k_obs_ or k_react_ (min^-1^)** | **Reference** |
| --- | --- | --- | --- | --- | --- | --- | --- | --- | --- |
| **Hth1307** | 1 μM | Ht ptRK^5:1^ | 2 nM | Yes | 1 mM | 150 mM | 37 | 0.58 | This work |
|  |  | Ht ptRK^10:1^ |  |  |  |  |  | 0.31 |  |
|  |  | Ht ptRK^14:1^ |  |  |  |  |  | 0.49 |  |
|  |  | Ht ptRK^20:1^ |  |  |  |  |  | 0.36 |  |
|  |  | Ht ptRK^5CCA^ |  |  |  |  |  | 0.05 |  |
|  |  | Tt ptRG^14:1^ |  | No |  |  |  | 0.008 |  |
|  |  | Sc ptRF^9:1^ |  |  |  |  |  | 0.006 |  |
|  |  | Bs ptRD^5CCA^ |  |  |  |  |  | 0.008 |  |
| **Aq880** | 50 nM | Tt ptRG^14^ | 5 nM | No  No | 4.5 mM | 20 mM | 37 | 0.62 | Feyh 2021 (1) |
|  | 500 nM |  |  |  |  |  |  | 2.06 |  |
| **Aq880** | varied | Tt ptRG^14^ | 1 nM | No | 4.5 mM | 20 mM | 37 | 1.43 | Nickel 2017 (2) |
| **At PRORP1** | 5 μM | Bs ptRD^1:1^ | 30 nM | No | 1 mM | 150 mM | 25 | 0.078 | Howard 2016 (3) |
|  |  | Bs ptRD^2:1^ |  |  |  |  |  | 0.15 |  |
|  |  | Bs ptRD^3:1^ |  |  |  |  |  | 0.032 |  |
|  |  | Bs ptRD^4:1^ |  |  |  |  |  | 0.025 |  |
|  |  | Bs ptRD^5:1^ |  |  |  |  |  | 0.025 |  |
|  |  | Bs ptRD^10:1^ |  |  |  |  |  | 0.025 |  |
|  |  | Bs ptRD^14:1^ |  |  |  |  |  | 0.02 |  |
|  |  | At mito-ptRC^5:1^ |  | Yes |  |  |  | 0.037 |  |
|  |  | At chlor-ptRF^5:1^ |  |  |  |  |  | 0.035 |  |
|  |  | At nuc-ptRC^5:1^ |  |  |  |  |  | 0.037 |  |
|  |  | At nuc-ptRF^5:1^ |  |  |  |  |  | 0.078 |  |
| **At PRORP2** |  | At mito-ptRC^5:1^ |  |  |  |  |  | 0.013 |  |
|  |  | At chlor-ptRF^5:1^ |  |  |  |  |  | 0.018 |  |
|  |  | At nuc-ptRC^5:1^ |  |  |  |  |  | 0.027 |  |
|  |  | At nuc-ptRF^5:1^ |  |  |  |  |  | 0.035 |  |
| **At PRORP3** |  | At mito-ptRC^5:1^ |  |  |  |  |  | 0.023 |  |
|  |  | At chlor-ptRF^5:1^ |  |  |  |  |  | 0.023 |  |
|  |  | At nuc-ptRC^5:1^ |  |  |  |  |  | 0.030 |  |
|  |  | At nuc-ptRF^5:1^ |  |  |  |  |  | 0.072 |  |
| **At PRORP1 ΔN76** | 5 μM | At mito-ptRC^5:1^ | 20 nM | Yes | 1 mM | 150 mM | 25 | 0.04 | Howard 2015 (4) |
|  |  | Bs ptRD^5:1^ |  | No |  |  |  | 0.03 |  |
| **At PRORP1 ΔN76** | 500 nM | At mito-ptRC^53:24^ | 25 pM | Yes | 1 mM | 150 mM | 25 | 1.4 | Howard 2012 (5) |
| **At PRORP2** | 5 μM | At nuc-ptRG^8:1^ | 50 nM | Yes | 1 mM | 150 mM | 25 | 1.1 | Karasik 2016 (6) |
|  |  | At nuc-ptRG^13:1^ |  |  |  |  |  | 0.7 |  |
|  |  | At nuc-ptRG^23:1^ |  |  |  |  |  | 0.7 |  |
|  |  | At nuc-ptRG^23:5^ |  |  |  |  |  | 1.0 |  |
|  |  | At nuc-ptRG ^23:10^ |  |  |  |  |  | 0.7 |  |
| **MRPP3** | 0.1-2 μM | Hs mito-ptRL^(UUR)6:1^ | 20 nM | Yes | 1 mM | 150 mM | 28 | 0.41 | Karasik 2019 (7) |
|  |  | Hs mito-ptRI^7:1^ |  |  |  |  |  | 0.8 |  |
|  |  | Hs mito-ptRM^6:1^ |  |  |  |  |  | 0.45 |  |
|  | 1.5 μM | Hs mito-ptRV^7:1^ |  |  |  |  |  | 0.3 |  |
|  |  | Hs mito-ptRS^(UCN)7:1^ |  |  |  |  |  | 0.06 |  |
|  |  | Hs mito-ptRS^(AGY)7:1^ |  |  |  |  |  | 0.06 |  |
| **At PRORP1** |  | Hs mito-ptRL^(UUR)6:1^ |  | No |  |  |  | 0.06 |  |
|  |  | Hs mito-ptRI^7:1^ |  |  |  |  |  | 0.05 |  |
|  |  | Hs mito-ptRM^6:1^ |  |  |  |  |  | 0.09 |  |
|  |  | Hs mito-ptRV^7:1^ |  |  |  |  |  | 0.08 |  |
|  |  | Hs mito-ptRS^(UCN)7:1^ |  |  |  |  |  | 0.69 |  |
|  |  | Hs mito-ptRS^(AGY)7:1^ |  |  |  |  |  | 0.09 |  |
| **At PRORP2** |  | Hs mito-ptRL^(UUR)6:1^ |  |  |  |  |  | 0.04 |  |
|  |  | Hs mito-ptRI^7:1^ |  |  |  |  |  | 0.03 |  |
|  |  | Hs mito-ptRM^6:1^ |  |  |  |  |  | 0.03 |  |
|  |  | Hs mito-ptRV^7:1^ |  |  |  |  |  | 0.03 |  |
|  |  | Hs mito-ptRS^(UCN)7:1^ |  |  |  |  |  | 0.31 |  |
|  |  | Hs mito-ptRS^(AGY)7:1^ |  |  |  |  |  | 0.04 |  |
| **At PRORP3** | 225 nM | Tt ptRG^7^ | trace amounts | No | 10 mM | 20 mM | 20 | 1.52 | Walczyk 2016 (8) |
|  | varied |  |  |  | 4.5 mM |  |  | 1.11 |  |
| **At PRORP1 ΔN76** | 2.5-22.5 μM | Bs ptRD^5^ | 30 nM | No | 20 mM | 330 mM | 25 | 2.62 | Teramoto 2020 (9) |

1. Feyh, R., Waeber, N. B., Prinz, S., Giammarinaro, P. I., Bange, G., Hochberg, G., Hartmann, R. K., and Altegoer, F. (2021) Structure and mechanistic features of the prokaryotic minimal RNase P. *eLife*. **10**, e70160

2. Nickel, A. I., Wäber, N. B., Gößringer, M., Lechner, M., Linne, U., Toth, U., Rossmanith, W., and Hartmann, R. K. (2017) Minimal and RNA-free RNase P in Aquifex aeolicus. *Proc. Natl. Acad. Sci.* **114**, 11121–11126

3. Howard, M. J., Karasik, A., Klemm, B. P., Mei, C., Shanmuganathan, A., Fierke, C. A., and Koutmos, M. (2016) Differential substrate recognition by isozymes of plant protein-only Ribonuclease P. *RNA*. **22**, 782–792

4. Howard, M. J., Klemm, B. P., and Fierke, C. A. (2015) Mechanistic Studies Reveal Similar Catalytic Strategies for Phosphodiester Bond Hydrolysis by Protein-only and RNA-dependent Ribonuclease P. *J. Biol. Chem.* **290**, 13454–13464

5. Howard, M. J., Lim, W. H., Fierke, C. A., and Koutmos, M. (2012) Mitochondrial ribonuclease P structure provides insight into the evolution of catalytic strategies for precursor-tRNA 5′ processing. *Proc. Natl. Acad. Sci.* **109**, 16149–16154

6. Karasik, A., Shanmuganathan, A., Howard, M. J., Fierke, C. A., and Koutmos, M. (2016) Nuclear Protein-Only Ribonuclease P2 Structure and Biochemical Characterization Provide Insight into the Conserved Properties of tRNA 5′ End Processing Enzymes. *J. Mol. Biol.* **428**, 26–40

7. Karasik, A., Fierke, C. A., and Koutmos, M. (2019) Interplay between substrate recognition, 5’ end tRNA processing and methylation activity of human mitochondrial RNase P. *RNA N. Y. N*. **25**, 1646–1660

8. Walczyk, D., Gößringer, M., Rossmanith, W., Zatsepin, T. S., Oretskaya, T. S., and Hartmann, R. K. (2016) Analysis of the Cleavage Mechanism by Protein-Only RNase P Using Precursor tRNA Substrates with Modifications at the Cleavage Site. *J. Mol. Biol.* **428**, 4917–4928

9. Teramoto, T., Kaitany, K. J., Kakuta, Y., Kimura, M., Fierke, C. A., and Hall, T. M. T. (2020) Pentatricopeptide repeats of protein-only RNase P use a distinct mode to recognize conserved bases and structural elements of pre-tRNA. *Nucleic Acids Res.* **48**, 11815–11826
